# Human-mediated secondary contact of two tortoise lineages results in sex-biased introgression

**DOI:** 10.1038/s41598-017-04208-4

**Published:** 2017-06-22

**Authors:** Eva Graciá, Roberto C. Rodríguez-Caro, Ana C. Andreu, Uwe Fritz, Andrés Giménez, Francisco Botella

**Affiliations:** 1 Ecology Area, Department of Applied Biology, Miguel Hernández University–Av. de la Universidad, Torreblanca, 03202 Elche Spain; 2 Department of Ecological Modeling, UFZ–Helmholtz Centre for Environmental Research, 04301 Leipzig, Germany; 3 Natural Processes Monitoring Team, Estación Biológica de Doñana (EBD-CSIC), c/Américo Vespucio s/n, 41092 Seville, Spain; 4 Museum of Zoology (Museum fürTierkunde), Senckenberg Dresden, A. B. Meyer Building, 01109 Dresden, Germany

## Abstract

Human-mediated secondary contact of recently diverged taxa offers valuable opportunities for studying the evolutionary mechanisms involved in the establishment and maintenance of genetic boundaries between taxa. We used mitochondrial and microsatellite markers to examine a recently introduced population of the spur-thighed tortoise (*Testudo graeca*) of mixed origin in the Doñana National Park (SW Spain). The earliest records of tortoises in Doñana trace back to the 18th century, but several population reinforcements in the 20th century with animals from Morocco are well-documented. Consequently, different genetic lineages, which represent distinct subspecies, are thought to co-exist there. Our results confirmed the presence of distinct lineages by revealing that tortoises of the subspecies *T. g. marokkensis* were introduced into a local allochthonous *T. g. graeca* population. Unexpectedly, *T. g. marokkensis* haplotypes exclusively appeared in males, and admixture levels were statistically sex-biased toward males. The sex ratio of the population deviated from parity, with males being 2.36-fold more abundant than females. Our results indicated that population reinforcements had a strong effect on the genetic composition of this population and aggravated its sex ratio deviation. We predict that this sex-biased pattern of introgression is ephemeral and advocated to the near loss of *T. g. marokkensis* haplotypes.

## Introduction

Species boundaries originate from limited or lacking gene flow between species caused by a set of intrinsic natural barriers. Introgression can be defined as the exchange and stable integration of alleles between genetically distinct populations and across such boundaries^1–3^. The resulting patterns, caused by hybridization and repeated back-crossing, largely depend on hybrid fitness and their ability to backcross with parental species^4, 5^. It has been widely demonstrated that introgression does not occur homogeneously within populations [e.g. refs 6–8]. Differences in mutation rates, selection pressures or demographic factors can cause cytonuclear discordance^9^ or sex-biased levels of introgression^1^. Cytonuclear discordance, defined as a significant difference between mitochondrial and nuclear genomes, is expected since the mitochondrial genome is inherited maternally in most species and has a fourfold smaller effective population size than the nuclear genome^10, 11^, which results in the quicker loss of ancestral polymorphisms over time^12^. Cytonuclear discordance is favored by mainly male-mediated gene flow, leading to sex-biased introgression. Sex-biased introgression is also often explained by Haldane’s rule, which states that “when in the offspring of two different animal races one sex is absent, rare, or sterile, that sex is the heterozygous (heterogametic) sex”^13^. Alternatively, differences in dispersal capabilities between males and females, or in selective pressures, can also promote such patterns^1^.

Abbott *et al*.^2^ recently pointed out that introgression could either promote or retard divergence processes, and vastly varies among hybridizing taxa and their divergence stages. In recently diverged groups, genetic boundaries are often subtle and allow gene flow by incomplete reproductive isolation. This is often the case in introductions that cause secondary contacts of recently diverged groups [e.g. refs 14–16]. Thus environmental disturbances and introductions of wild or domestic taxa are valuable opportunities to study the evolutionary mechanisms involved in the establishment and maintenance of genetic boundaries between taxa. However, these situations are not desirable from a conservationist point of view. Exchange of genes between recently isolated lineages could result in loss of local adaptations^15^, or the transmission of genes from domestic to wild populations can even threaten natural processes in ecosystems (e.g. exchange of genes conferring herbivory resistance^17^).

**Figure 1:**
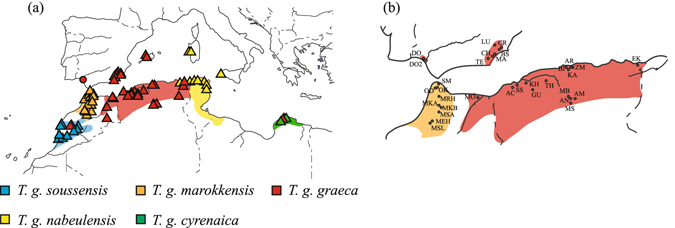
(**a**) Approximate distribution of the subspecies of the spur-thighed tortoise (*Testudo graeca*) that correspond to different genetic lineages (North Africa and western Europe). Triangles refer to sites with cyt *b* sequences available from GenBank, while the circle indicates previously published partial cyt *b* sequences^23^ for the Doñana population. The distribution range of a sixth lineage, recently discovered in Libya, remains unknown^22^. (**b**) Sampling design for genotyping with microsatellites. Complete names of sampling sites are shown in Supplementary Table S1. Maps were modified from Graciá *et al*.^22^ using Adobe Illustrator CS6 (www.adobe.com/Illustrator).

Here we address introgression patterns by examining the origin and historic management of the spur-thighed tortoise, *Testudo graeca* Linnaeus, 1758, in southwest Spain, which putatively led to the admixture of relatively recently diverged genetic lineages from North Africa. The spur-thighed tortoise is one of the most widely distributed tortoise species in the world. Its western Palearctic distribution range embraces North Africa, the Middle East, Asia Minor and southeast Europe. Moreover, a few small isolated populations occur at two distant sites in southwest and southeast regions of the Iberian Peninsula, and also on Majorca, Sardinia and Sicily^18, 19^. In North Africa, a monophyletic group of six mitochondrial lineages shows a west-east vicariant diversification pattern with allo- and parapatric ranges (Fig. 1a)^20–22^. Meanwhile, only one mitochondrial lineage per population is present on the European side of the Western Mediterranean, whose populations generally result from historical introductions from North Africa^20, 22–24^ (Fig. 1a). Among them, the population in southeastern Spain has been identified as the most ancient as it dates back to around 20,000 years ago, but is also derived from North African founders^25^. Despite its iconic location in the Doñana National Park (southwest Spain; referred to as “Donaña” henceforth), the other population that inhabits the Iberian Peninsula remains genetically unstudied.

**Figure 2:**
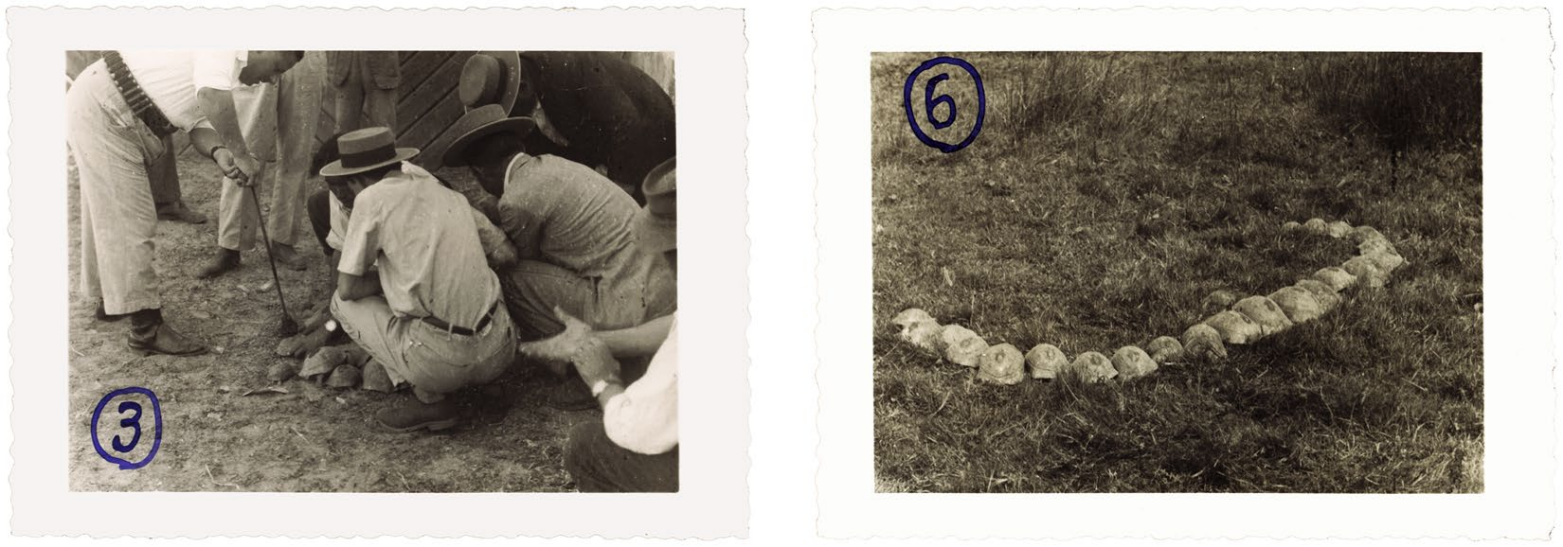
Photographic record of the spur-thighed tortoise population reinforcements in Doñana in the last century. Numbers refer to a set of 12 photographs that document introductions of tortoises from Morocco: (3) iron branding of tortoises; (6) release of 20 marked tortoises in the “Sopetón” area of the reserve.

The earliest references of tortoises that inhabit Doñana trace back to the 18th century. Historical texts mention *expressis verbis* “icotecas” and “galapagos” to specifically differentiate between tortoises and turtles^26^. It has also been well documented that this population was repeatedly reinforced using tortoises from Morocco throughout the 20th century, as illustrated by some early photographs that document the history of this National Park (Fig. 2). In particular, Valverde^27^ estimated that less than 100 Moroccan spur-thighed tortoises were introduced into the reserve between 1948 and 1954. The single genetic study, which addressed the origin of *T. graeca* in Doñana, included four samples from this population and was based exclusively on mitochondrial data. It found a shared lineage for Doñana, southeastern Spain, Majorca, east Morocco and northern Algeria^23^. This lineage is currently identified with the subspecies *T. g. graeca* ^20^. Based on this finding, the hypothesis that *T. graeca* arrived in the Iberian Peninsula before the Strait of Gibraltar opened could be rejected, which agrees with the fact that no fossil record exists^28^.

By considering the geographical location of the Doñana population, its origin could be either explained by (i) introduction or, alternatively, (ii) by it representing a relic from a formerly continuous range across southern Spain^29^. Furthermore, as very few individuals from the Doñana population have been studied to date, the effects of recent introductions of tortoises from Morocco could have been overlooked. Apart from *T. g. graeca*, two other subspecies (*T. g. marokkensis* and *T. g. soussensis*; Fig. 1a) occur in Morocco, both with much wider distribution ranges than *T. g. graeca*. Thus their genetic signatures could be detectable when more tortoises from Doñana are studied.

To elucidate this situation in more detail, the specific aims of the present study were to: (i) explore the genetic impact that the recent species management could have had; (ii) characterize the origin of the spur-thighed tortoises in Doñana. To do so, we analyzed 85 spur-thighed tortoises from Doñana by sequencing a mitochondrial DNA fragment (cyt *b*) and genotyping them at eleven microsatellite loci. These molecular data were analyzed together with the available cyt *b* sequences for all the North African lineages (Supplementary Table S1)^20, 22, 24, 25, 30^ and genotypes for all the populations potentially related with the tortoises from Doñana (southeastern Spain and North Africa; Fig. 1b).

Toward our first aim, we calculated the proportion of the different mitochondrial haplotypes in Doñana and inferred their lineage. Mismatch distributions^31^ served to identify the signatures of recent population reinforcements. We also calculated individual admixtures by a Bayesian clustering approach^32^. We estimated the biases between mtDNA and microsatellite data, and assessed sex-biased levels of introgression. Sex-biased introgression was not expected because *Testudo graeca* is known to have temperature-dependent sex determination, like the majority of chelonians^33, 34^, and because we used neutral genetic markers.

Toward our second aim, we expected genetic signatures for a recent origin, in particular low genetic variation and weak divergence from the source population, as previously found in other western European *T. graeca* populations^22, 24, 25^. We inferred these patterns from population genetic descriptors and Bayesian cluster analyses. Then we calculated mismatch distributions^31, 35^ and neutrality indices^36, 37^, which could reflect the signatures of demographic expansions using mitochondrial data. Microsatellites can also provide evidence for departures from stability, including Hardy-Weinberg and linkage disequilibrium, or heterozygosity excess at selectively neutral loci as a result of loss of low-frequency alleles^38, 39^. Apart from these analyses, we used Bayesian demographic approaches^40, 41^ to reconstruct the diversification history of the Doñana population prior to the reinforcements with Moroccan tortoises.

## Results

### The *Testudo graeca* lineages from Doñana

**Figure 3:**
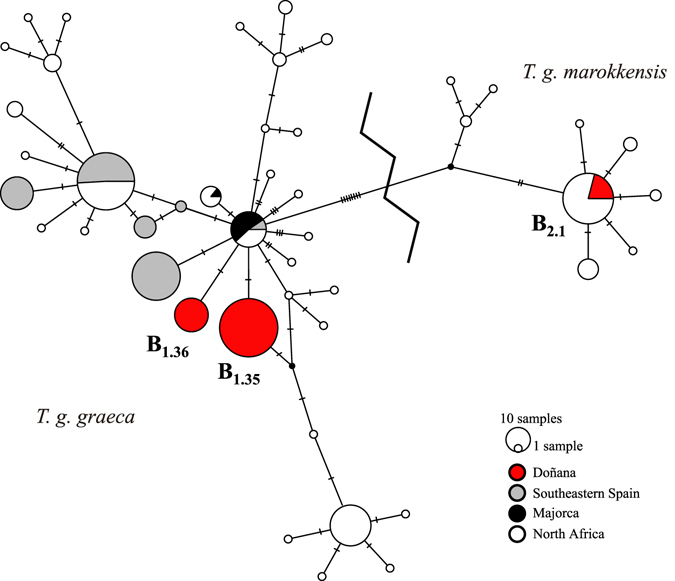
Parsimony network for cyt *b* haplotypes from Doñana, *Testudo graeca graeca* and *T. g. marokkensis*. Colors indicate the proportion of haplotypes found in Doñana, southeastern Spain, Majorca and North Africa. Dashes across lines indicate one nucleotide substitution. Only those haplotypes mentioned in the text are highlighted.

**Figure 4:**
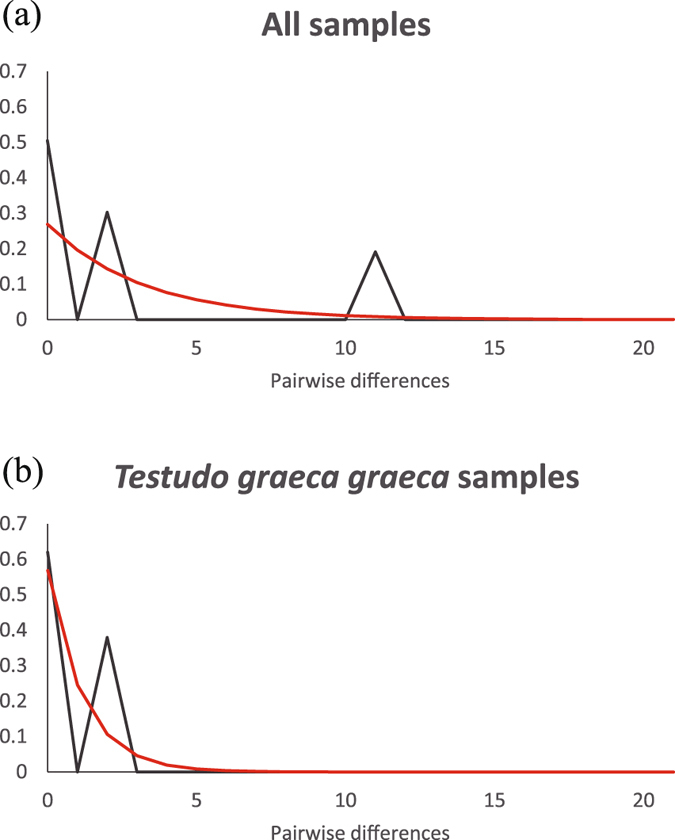
Demographic history of the spur-thighed tortoises (*Testudo graeca*) in Doñana, as determined from the mismatch distributions for: (**a**) all the haplotypes found in Doñana (including the haplotypes of the two lineages *T. g. graeca* and *T. g. marokkensis*); (**b**) only considering the original *T. g. graeca* haplotypes. Gray lines correspond to the observed data and red lines to the expected data for constant population sizes.

The 85 cyt *b* sequences from Doñana corresponded to three different haplotypes (Supplementary Table S1). Two abundant unique haplotypes were recorded for the first time. Both corresponded to the *T. g. graeca* lineage (B_1.35_ and B_1.36_, shared by 57 and 19 of the individuals, respectively; GenBank accession numbers: LT838800 and LT838801, respectively). Another haplotype was present in nine males, which represented the lineage of *T. g. marokkensis* (B_2.1_). This haplotype is widely distributed in northern Morocco (Fig. 3). A parsimony network connected all the haplotypes using the 95% criterion. The two unique haplotypes from Doñana differed by two mutational steps, and by one step from the B_1.2_ haplotype that occurs in North Africa, southeastern Spain and Majorca (Fig. 3 and Supplementary Table S1). A mismatch distribution also supported the co-existence of different lineages in Doñana, as indicated by two markedly different peaks (*rg* = 0.67; *p* = 0.99; Fig. 4a). The lesser frequency of the *T. g. marokkensis* haplotypes supported the notion that this lineage was introduced into Doñana during the twentieth-century population reinforcements.

**Figure 5:**
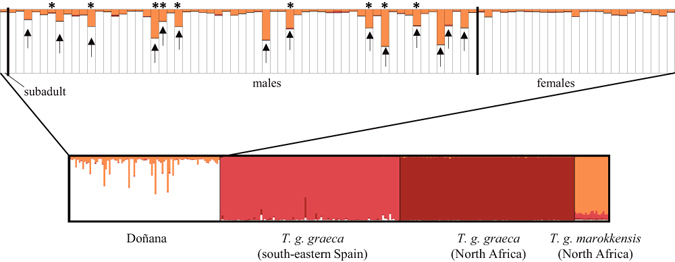
Structure bar plots using sampling groups as prior information and with the cluster number set at four. Vertical bars represent spur-thighed tortoises (*Testudo graeca*), while the amount of each color indicates the proportion of each inferred cluster. For more details, the admixture levels of the Doñana individuals are zoomed. Asterisks indicate those tortoises that harbor the mitochondrial haplotypes of *T. g. marokkensis*, while arrows denote those with memberships to the *T. g. marokkensis* cluster >0.125.

Subsequent analyses using microsatellites considered *T*. *g*. *graeca* from North Africa (*n* = 99) and southeastern Spain (*n* = 102), as well as *T*. *g*. *marokkensis* from Morocco (*n* = 19). These markers not only corroborated the existence of two different lineages in Doñana, but also the admixture between them. One locus (GP81) proved monomorphic. For the remaining ten, 110 alleles were found, ranging from two (Test76, Test96) to 28 alleles (GmuD51). No significant linkage disequilibrium was revealed for any of the 55 pair-wise locus combinations, and none locus showed at sampling sites significant homozygote excess indicating the presence of possible null alleles. After these initial analyses, we used a cluster number (*K*) of four in Structure analyses^32^, with prior group information that corresponded to (1) Doñana, (2) *T. g*. *graeca* from southeastern Spain, (3) *T. g. graeca* from North Africa (Algeria and Morocco) and (4) *T. g. marokkensis* from North Africa (Morocco). The analysis supported the differentiation of these four groups. The Doñana samples were assigned mostly to a specific cluster. Of the 85 analyzed samples, 14 males showed consistent assignment values over 0.125 to the North African *T*. *g*. *marokkensis* group that were, therefore, identified as having mixed ancestries of *T*. *g*. *graeca* and *T*. *g*. *marokkensis*. These individuals corresponded to eight of the nine tortoises that harbored the mitochondrial haplotypes of *T*. *g*. *marokkensis*. Not one single female with mixed ancestries was found, and no sample showed consistent mixed ancestry with any other group (Fig. 5). The 14 samples with mixed ancestries from Doñana and the *T*. *g*. *marokkensis* samples from Morocco were removed from subsequent analyses to explore the origin of the original *T*. *g*. *graeca* Doñana population (i.e. prior to the population reinforcements).

### Genetic differentiation of *T. g. graeca* in Doñana

**Figure 6:**
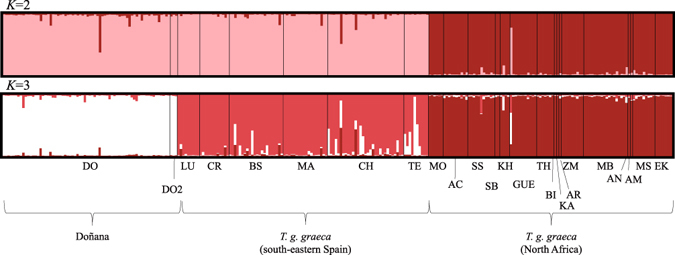
Structure bar plots for *K* = 2 and *K* = 3. The Moroccan samples of *Testudo graeca marokkensis* and those from Doñana with memberships to this lineage >0.125 were excluded. Locations of sampling sites are shown in Fig. 1b. For further explanation, see Fig. 5.

Using only the pure *T. g. graeca* from Doñana, Structure analyses without prior population information revealed that *K* = 3 was the optimal cluster number, although *K* = 2 also yielded a high Δ*K* value (*K* = 2: Δ*K* = 171.8; *K* = 3: Δ*K* = 240.58; *K* = 4: Δ*K* = 4.59; Supplementary Fig. S1). Different runs were consistent and showed moderate levels of admixture among clusters. For *K* = 2, all the North African individuals were placed in one cluster and all the Spanish tortoises in the second. Higher resolution was achieved for *K* = 3 with the individuals from the two Iberian populations divided into two clusters (Doñana and southeastern Spain; Fig. 6). In the latter, 93% of the tortoises from Doñana, 74.5% of those from southeastern Spain, and 95% of the tortoises from North Africa were assigned to their respective clusters with membership probabilities of >0.9.

Pair-wise *F*_ST_ values among the sampling sites were generally significant (Supplementary Table S2). The weakest differentiation (*F*_ST_ = 0.002) was found between the sites Guetourfa (GU) and Messad (MS) in Algeria, while the highest value (*F*_ST_ = 0.37) was observed between a site in the northern Molouya Valley (MO) in Morocco and another site in the north of the southeastern Spanish range (Crisoleja; CR). The lowest *F*_ST_ values (0.079 for both) for the Doñana population were found for two southeastern Spanish sites: south of the Bas Mountain (SB) and Chinas (CH).

Hierarchical AMOVAs supported the two-cluster solution of Structure. A more pronounced differentiation was revealed for *K* = 2, with 16.74% of global genetic variance between the Iberian and North African *T. g. graeca*, 7.73% among the sampling sites within clusters, and 11.61% that corresponded to the variance among individuals within sites (*F*_RT_ = 0.17; *F*_SR_ = 0.09; *F*_ST_ = 0.25; *F*_IS_ = 0.15; *F*_IT_ = 0.36; all *p* = 0.001). For *K* = 3, 12.99% of global variance occurred among the two Iberian and the North African populations, 7.4% among sampling sites, and 12.25% among individuals (*F*_RT_ = 0.13; *F*_SR_ = 0.09; *F*_ST_ = 0.2; *F*_IS_ = 0.15; *F*_IT_ = 0.32; all *p* = 0.001).

### Genetic diversity and inference of recent demographic processes for *T. g. graeca* in Doñana

The *T. g. graeca* tortoises from Doñana showed lower levels of genetic diversity for cyt *b* than the North African and southeastern Spanish representatives of the same genetic lineage, but higher levels compared to those from other recently introduced western European populations (Table 1). The mismatch distribution of the *T. g. graeca* haplotypes did not reveal any signs of recent demographic expansion (*rg* = 0.67; *p* = 0.89; Fig. 4b). In the same vein, the Tajima’s *D* and Fu’s *F*_*S*_ values yielded positive values, but neither revealed a significant deviation from zero (*D* = 1.40*; p* = 0.92; *F*_S_ = 3.34; *p* = 0.95).

**Table 1:** Estimates of the haplotype (*h*) and nucleotide (π) diversities for the North African and western European samples (standard deviations in brackets).

Geographic area	Subspecies	N	*h*	*π*
Doñana (southwest Spain)	*T. g. graeca*	76	0.38 (0.05)	0.0006 (0.0008)
	*T. g. marokkensis*	9	0	0
Southeastern Spain	*T. g. graeca*	97	0.72 (0.02)	0.0023 (0.0006)
Majorca (Spain)	*T. g. graeca*	12	0.17 (0.13)	0.0001 (0.0001)
Sardinia	*T. g. nabeulensis*	31	0.07 (0.06)	0.0001 (0.0001)
Sicily	*T. g. nabeulensis*	1	—	—
North Africa	*T. g. nabeulensis*	36	0.80 (0.04)	0.0013 (0.0002)
	*T. g. graeca*	106	0.85 (0.02)	0.0030 (0.0002)
	*T. g. marokkensis*	52	0.56 (0.08)	0.0009 (0.0002)
	*T. g. soussensis*	49	0.80 (0.04)	0.0029 (0.0002)
	*T. g. cyrenaica*	4	0.67 (0.20)	0.0012 (0.0009)

The microsatellite data also supported a stable population scenario for Doñana. The allelic diversities between the two Iberian populations were similar, but were slightly lower than for the North African *T. g. graeca*. The expected heterozygosity levels were similar across the three populations and all the *F*_*IS*_ values came close to zero (which indicates a balance between the expected and observed heterozygosities; Table 2). After Bonferroni correction, all the loci corresponded to Hardy-Weinberg equilibrium expectations for Doñana. The heterozygosity tests did not reveal signatures of heterozygosity excess or deficiency for this population (SMM: *p* = 0.46; TPM: *p* = 0.95). Consequently, no recent demographic bottlenecks or expansions were inferred for the Doñana population.

**Table 2:** Descriptors of genetic diversity in the groups detected by Structure for clustering solutions *K* = 2 and *K* = 3.

Structure analysis	Cluster solution	Cluster	*N*	*N*_A_	*A*_E_	*H*_O_	*uH*_E_	*F*_IS_
Without prior information	*K* = 2	Doñana (*T. g. graeca*) + southeastern Spain	173	6.34	3.48	0.37	0.39	0.060
		North Africa	99	9.18	4.42	0.37	0.40	0.131
Sampling location as prior information	*K* = 3	Doñana (*T. g. graeca*)	71	5.18	3.30	0.39	0.38	−0.004
		Southeastern Spain	102	5.27	2.52	0.35	0.37	0.371
		North Africa	99	9.18	4.42	0.37	0.40	0.131

*N*, number of genotyped individuals; *N*_A_, mean number of alleles; *A*_E_, mean number of effective alleles; *H*_O_, mean observed heterozygosity; *uH*_E_, mean unbiased expected heterozygosity; *F*_IS_, mean inbreeding coefficient.

### Historical divergence and past demographic inference of *T. g. graeca* in Doñana

**Figure 7:**
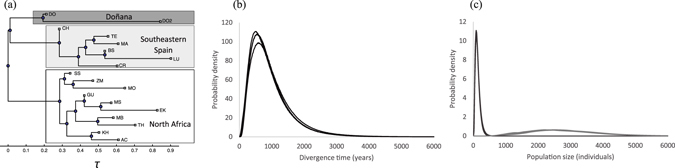
(**a**) Estimated population history for the *Testudo graeca graeca* populations inferred and plotted using Bananas ^41^. (**b**,**c**) Subsequent analyses using IMa2^40^ served to date the split between DO and CH as being representive of the Doñana and southeastern Spain populations. Lines represent probability density estimations of four independent replicates of the divergence time parameter (**b**), and of population sizes (**c**) for Doñana (dark gray) and southeastern Spain (light gray). Locations of sampling sites are shown in Fig. 1b.

The historical order of divergence inferred from Bananas ^41^ matched the genetic differentiation revealed by Structure (Fig. 7a). The binary rooted tree topology assigned the populations from North Africa to a single group, which identified the populations from Doñana and southeastern Spain as sister populations.

In order to set a date for the origin of *T. g. graeca* in Doñana, we investigated its demographic history using IMa2^40^. For these analyses, we considered the Doñana (DO) and Chinas (CH) sites as representative for Doñana and southeastern Spain, which were characterized by the lowest pair-wise *F*_ST_ values. All the effective sample size (ESS) values for the estimated parameters exceeded 100 at the different replicates, which indicates sufficient Markov chain Monte Carlo mixing across the parameter space^42^. The marginal posterior probability densities of the demographic parameters did not differ among replicates. The split between Doñana and southeastern Spain was estimated to be 947.5 years, with 95% highest posterior density intervals (HPDI) of 200.8–2,599.5. The effective Doñana population size was estimated at 136.3 individuals (95% HPDI: 40.5–323.9) and at 2,816.8 individuals (95% HPDI: 1,048.25–5,322.8) for southeastern Spain. The plots of the marginal posterior probability densities of the demography parameters are shown in Fig. 7b,c.

### Tests for sex-biased introgression and cytonuclear discordance of the spur-thighed tortoises from Doñana

The Structure results for the microsatellite data indicated signatures of admixture between *T. g. graeca* and *T. g. marokkensis* in 16.6% of our samples. We did not find signatures of cytonuclear discordance as 57.14% of these samples harbored the mitochondrial haplotype of the *T. g. marokkensis* lineage.

However, and unexpectedly, not a single female from Doñana was identified as having a mixed ancestry according to the microsatellite data, and no female tortoise harbored the allochthonous *T. g. marokkensis* haplotype. These sex-biased distributions were significant according to the chi-square tests (for mtDNA: χ^2^ = 8.05; df = 3; *p* = 0.045; for microsatellites = χ^2^ = 11.93; df = 3; *p* = 0.008). Based on our sampling, the sex ratio of the population deviated from parity, and males were 2.36-fold more abundant than females (*N*_*females*_ = 25; *N*_*males*_ = 59; *N*_*subadults*_ = 1; *p* < 0.001). After removing the 15 males with mixed genotypes or *T. g. marokkensis* haplotype (14 from Structure and one from mtDNA analyses), the population’s sex ratio remained skewed (*p* = 0.03).

## Discussion

In the present study, we provide evidence for introgression signatures between two recently diverged lineages of spur-thighed tortoises that co-exist in Doñana as a result of introductions and historical management. In the Doñana population we found a major proportion of haplotypes and genotypes that represented *T. g. graeca*, which undoubtedly constitute the original genetic lineage in Doñana. However, approximately 15% of tortoises had a genetic impact from *T. g. marokkensis*, the subspecies that was introduced in the past century as part of population reinforcements.

Our results reveal no population expansion or bottleneck signatures, but indicate demographic stability for the original population, but also corroborated a recent origin of the Doñana population. Compared to the southeastern Spanish *T. g. graeca*, a weak genetic differentiation appears, which suggests this population as the source of the tortoise population in Doñana. The southeastern Spanish *T. g. graeca* originated, in turn, from North African founders in prehistorical times, and the role that humans could have played as potential dispersers is still a matter of debate^20, 25, 43, 44^.

According to our results, the populations from Doñana and southeastern Spain are very closely related. The low genetic differentiation between them and their young historical divergence definitively rule out the possibility that both represent vestiges of an ancient wider distribution in the Iberian Peninsula, as previously suggested^29^. By considering the wider distribution range and the effective population size of the species in southeastern Spain, our results instead suggest that *T. g. graeca* was introduced into Doñana with founder individuals from southeastern Spain. In Doñana there are several examples of introduced species, including the recent introduction of fish (e.g. *Gambusia holbrooki* or *Cyprinius carpio* ^45^) or game species (e.g. *Dama dama* ^46^). Other examples include ancient introductions of chameleons (*Chamaeleo chamaeleon*) or genets (*Genetta genetta*), which may be related to Phoenicians or Arabs (some 3,100–500 years ago)^47, 48^. Further historical and archaeological research is needed to gain a better understanding of these and other historical introductions across the Strait of Gibraltar because based on molecular data analyses alone, minor inaccuracies in calibration could dramatically change date estimates.

The present study also provides firm evidence for the intrinsic mechanisms that cause a male excess in the Doñana population as a result of exogamy. This population’s sex ratio has been traditionally described as significantly skewed toward males, with ratios of up to 3.32:1 adult males:adult females^26^. In contrast, the majority of the *T*. *graeca* populations show balanced sex ratios [e.g. refs 49–51], and very few studies have reported deviations from a balanced sex ratio for the genus *Testudo*. In these cases, sex ratios skewed toward males correlate positively with high population densities and aggressive courtship behavior (*T. hermanni* in Greece^52, 53^) or with an over-collection of larger females (*T. graeca* in Morocco^49^). In Doñana, highly admixed genotypes occur exclusively in males. However, one male with a weak admixed genotype harbors a *T. g. marokkensis* haplotype (Fig. 5), which suggests that although admixed females occur, they are rare. Sex-biased introgression is often explained by Haldane’s rule^13^, which requires heterogametic sex chromosomes. This pattern is not expected in *T. graeca* because it has no sex chromosomes and temperature-dependent sex determination^32, 33^. Alternatively, other mechanisms may explain the observed lack of admixed females: first, the impact of exogamy on the sex determination process; second, sex-biased selection in favor or against admixed individuals. In the former, changes in threshold temperatures to determine sex^33^ could bias the determination of clutches of hybrids to favor males. Previous studies have examined *T. graeca* reproduction in Doñana and have found highly variable hatching success rates, incubation temperatures and incubation periods between different nesting periods and years^54, 55^. Part of this variation could be caused by the admixture of both lineages, which would render this population as being particularly interesting for studying the mechanisms that underlie sex determination in *T. graeca*. The epigenetic mechanisms that govern temperature-dependent sex determination are largely unknown^56, 57^, and exogamy may affect it. Rarity of admixed females can be explained alternatively by lower female survival due to either genomic constraints (endogenous selection) or their poor adaptation to local environmental conditions (exogenous selection)^58^. In a similar vein, better fitness of hybrid males compared to pure males, known as the heterosis effect or hybrid vigor^59^, would increase the genetic signatures of the admixed males in the population. Long-term demographic studies in the Doñana have found that adult survival rates are high, constant and similar between males and females, while recruitment and juvenile survivals are variable. These studies have also observed a negative population trend over the 1980–1985 period, while a positive trend has been found for 1992–1995^26, 60^. Continuous population monitoring, combined with parallel genetic analyses, would help to elucidate whether exogamy impacts adult or early life stages, and if is related to the negative population dynamics reported after population reinforcements.

Thus the observed male-biased sex ratio is a prelude of future and stable cytonuclear discordance in which *T. g. graeca* haplotypes will be fixed, but *T. g. marokkensis* alleles will persist across nuclear genomes. Similar patterns for the species have been reported in both eastern Algeria, in the secondary contact zone of *T. g. graeca* and *T. g. nabeulensis* ^25^; and Transcaucasia, in the contact zone of *T. g. armeniaca*, *T. g. buxtoni* and *T. g. ibera* ^61, 62^. To date, these patterns have been explained by the low dispersal capabilities of females^61, 62^. However, the present study suggests that exogamy could promote sex ratio deviations, which would play a role in maintaining the genetic integrity of genetic lineages across the species’ range^1^.

Finally despite its anthropogenic origin, the Doñana population constitutes a valuable empirical system for studying the evolutionary mechanisms that underlie introgression. Our findings also underline the need to carefully manage wild tortoise populations. Unexpected consequences of inadequate introductions or translocations may threaten the viability of wild populations, especially if they have low densities.

## Methods

### Sampling and available sequences considered

Two field surveys were conducted in autumn 2012 and spring 2013. Two sites were studied in the Doñana National Park, which were 12 km apart. Sixty-five tortoises were studied from “El Puntal” (DO) and four from “Marismillas” (DO2). Adult tortoises were sexed according to external secondary characters, while one subadult was treated as not sexable^26^. Sixteen additional samples with known sex were provided by the Doñana Biological Station, which were used in a previous study to address multiple paternity and sperm storage in *T. graeca* ^63^. In summary, 85 samples from Doñana were analyzed.

In order to represent the probable sources of *T. graeca* in Doñana, our previously accumulated data set of 396 sequences from the western Mediterranean clade was used. The considered mitochondrial DNA fragment comprised the complete mitochondrial cytochrome *b* gene and 25 bp of the adjacent tRNA-Thr gene (1164 bp). This data set corresponded to 250 samples from North Africa, 97 from southeastern Spain, 36 from Sardinia, 12 from Majorca and one from Sicily, which were analyzed for previous studies (Fig. 1a; Supplementary Table S1)^20, 22, 24, 25, 30^. After identifying the lineages present in the Doñana, the Dresden Zoology Museum and the Miguel Hernández University collections provided additional samples that are representative of their potential sources for their genotyping with microsatellites (Fig. 1b). In addition to the Doñana samples, the final data set of genotypes (*n* = 305) included samples from six southeastern Spanish sites (subspecies *T. g. graeca*; *n* = 102) and from 26 sites from Morocco and Algeria (subspecies *T. g. graeca* and *T. g. marokkensis*; *n* = 99 and *n* = 19, respectively). No samples from Majorca were considered because the origin of this population is very recent and is related directly with Algerian populations^22^.

### Laboratory procedures

Total genomic DNA was isolated from an ethanol-preserved blood samples by the salt-extraction protocol^64^. Cyt *b* sequences were amplified by polymerase chain reaction (PCR) and subsequently sequenced. For this purpose, previously described primers (CytbG^65^, mt-c-For2, mt-f-na3 and mt-E-Rev2^66^) and PCR conditions^66^ were used. PCR products were purified by precipitation in 1 volume of PCR product (30 μL), 1 volume of 4 M NH_4_Ac (30 μL) and 12 volumes of EtOH (100%; 360 μL). DNA was pelleted by centrifugation, washed with 70% ethanol and subsequently dissolved in 20 μL of H_2_O. PCR products were sequenced in an ABI 3130xl sequencer (Applied Biosystems, Foster City, CA, USA).

Tortoises were genotyped using eleven fluorescent-labeled microsatellites^43^: GmuB08^HEX^, GmuD16^NED^, GmuD51^PET^ (designed for *Glyptemys muhlenbergii* ^67^), GP55^HEX^, GP61^HEX^, GP81^6-FAM^, GP96^6-FAM^ (designed for *Gopherus polyphemus* ^68^), Test10^6-FAM^, Test21^6-FAM^, Test71^6-FAM^ and Test76^HEX^ (designed for *Testudo hermanni* ^69^). Microsatellite loci were individually PCR-amplified in a final volume of 20 *μ*l using 1 unit of *Taq* polymerase (Biotaq, Ecogen, Spain) with the buffer recommended by the supplier and a final concentration of 1.6 mM MgCl_2_, 0.2 mM of each dNTP (dNTPs Mix, Ecogen, Spain), 0.4 *μ*M of each primer, and approximately 10–40 ng of total DNA. PCR products were analyzed by gel electrophoresis in two separate runs (first run: Test71, Test10, Gp61, Gp81, Gp55; second run: Test76, Test96, Test21, GmuD16, GmuB08, GmuD51) in an ABI 3130xl sequencer and with the GeneMapper software (Applied Biosystems).

### Assessing introgression due to recent population reinforcements in Doñana

The mitochondrial cyt *b* sequences were aligned with ClustalW using the default parameters as implemented in Mega 4.0^70^. The identified new haplotypes were deposited in GenBank and named following the nomenclature of previous works^20, 22, 24, 25^. The mitochondrial DNA variation of the Doñana population was characterized by constructing a parsimony network because this approach is especially effective when representing phylogenetic relationships within closely related taxa^71^. Networks were built using Tcs ^72^. To detect the genetic signatures of introgression in Doñana population, we calculated and plotted a mismatch distribution using cyt *b* sequences with Dnasp ^73^. With an infinite sites model, stable populations would be depicted by stochasticity as ragged and multimodal distributions, whereas introgression processes would usually be reflected in bimodal distributions. The raggedness index *rg* was considered to test the fit of the observed data to the expected distribution^74^. The impact of the recent population reinforcements in the Doñana population was quantified by calculating the percentages of the individuals that pertained to different lineages. It was assumed that the lineage with a much smaller proportion was that introduced in the last century. To detect any biases in the percentage of introduced haplotypes between sexes, Chi-square tests were used.

Microsatellite loci were examined for null alleles using Micro-Checker 2.2.3^75^. Genotypic linkage disequilibrium between pairs of microsatellite loci was tested by the Markov chain Monte Carlo (MCMC) method implemented in Genepop 4.0^76^. Tortoise ancestry was examined by the Bayesian clustering approach of Structure 2.3.4^32^. *K* = 4 was fixed by considering four groups: Doñana, southeast Spanish *T. g. graeca*, North African *T. g. graeca* and North African *T. g. marokkensis*. These groups were used as priors for the ‘locprior’ option^77^. Burn-in was set at 10^5^ and the number of iterations was set at 10^6^. The individuals from Doñana with cluster membership proportions of >0.125 to any of the other groups were treated as having mixed ancestries (corresponding to a single great-grandparent over three generations). To evaluate the convergence of cluster memberships across individuals, 10 replicates of the analysis were run. As mentioned before for the mitochondrial data, Chi-square tests for independence were used to detect any biases in the percentages of haplotypes between sexes.

### Genetic diversity, differentiation and potential sources of *T. graeca* in Doñana prior to population reinforcements

Microsatellite data and the Structure software also served to explore the genetic differences in the Doñana population and its potential source sites in southeastern Spain and North Africa (eastern Morocco and northern Algeria). For this purpose, Structure was run with and without prior population information from *K* = 1 to *K* = 25 (total amount of collection sites, plus one) using the admixture model and the ‘correlated allele frequencies’ option. Burn-in was set at 10^4^ and the number of iterations was set at 10^5^. To evaluate convergence and to estimate optimal genetic clustering, 10 replicates were run for each *K* value. The number of populations that best fitted the data set was defined by the Δ*K* method^78^, as implemented in Structure Harvester ^79^. The assignment of genotypes to clusters was visualized using Distruct ^80^.

We calculated the haplotype (*h*) and nucleotide (*π*) estimates of diversity from the mitochondrial cyt *b* sequences using Dnasp ^73^. Genalex 6.5.01^81^ was used to calculate the descriptive statistics of genetic diversity from the microsatellite data in the genetic groups obtained from clustering: average number of alleles (*N*_A_), mean number of effective alleles (*A*_E_), average observed heterozygosity (*H*_O_), unbiased average expected heterozygosity (u*H*_E_) and inbreeding estimates (*F*_IS_), and to test for Hardy-Weinberg deviations. This software was also used to calculate the pair-wise *F*_ST_ values as measures of genetic differentiation among the considered sites, and hierarchical AMOVAs were run to determine how genetic variation was distributed among clusters, sites and individuals. In the calculation of the pair-wise *F*_ST_ values, only the sampling sites with at least five genotyped individuals were considered.

### Historical divergence and the past demographic history of *T. graeca* in Doñana prior to population reinforcements

The mismatch distributions from the cyt *b* sequences served to detect the genetic signatures of past demographic expansions in the Doñana population. Mismatch distributions were calculated and plotted with Dnasp, and tested using the raggedness index *rg*. In the mismatch distributions, expansion processes were expected as unimodal distributions. In addition to mismatch distributions, the *F*_*S*_^36^ and *D* ^37^ statistics were calculated in Dnasp using the cyt *b* data. Significantly negative values indicate excess rare alleles, which was expected according to recent demographic expansions. Contrarily, positive values indicate the elimination of rare alleles by recent bottleneck events. These analyses were carried out according to selective neutrality and population equilibrium assumptions, and their significance was assessed with 1000 coalescent simulations.

The demographic and diversification history of the Doñana population was also inferred using microsatellite data. Bottleneck 1.2.02 ^39^ served to test current excess or defect of heterozygosity, which was likely to arise from recent population size reductions or expansions. The step-wise mutation model (SMM) and the two-phase mutation model (TPM) with 78% step-wise mutations *(p*_*s*_), 22% multistep mutations (*p*_*g*_), and a variance of 3.1 among multiple steps, as recommended for microsatellite data^82^, were used. Then the historical divergence of the sampling sites was inferred from the pair-wise analysis in the Bananas software^41^. This approach allowed us to identify the sister population of the Doñana population. Bananas uses Bayesian inference, and the Adaptive Metropolis Algorithm as an adaptive Markov chain Monte Carlo sampler, to infer Neighbor Joining trees in which branch lengths represent mean diversification times *τ* (*τ* = *t*/*N*; being *t* the number of generations and *N* the population size) among populations. The model is based on a diffusion approach to the transition density of a neutral infinite alleles Wright-Fisher model. The number of iterations was set at 10^5^, burn-in at 10^4^, and thinning at 10. Only those sampling sites with at least three genotyped samples were considered in this analysis. Finally, the split between the Doñana population and its sister population was estimated using the IMa2 software^40^. Four independent replicates were performed to simultaneously estimate divergence times (*t*) and effective population sizes (*Ne*). The Step-wise Mutation Model was assumed and a run length of 2 × 10^6^ steps was computed after a burn-in of 10^5 ^steps. The upper bounds of the prior distributions for parameters were set at *t* = 0.1; *qa* = 10 (effective ancestral population size); *q1* and *q2* = 0.1 (effective sizes of the Doñana and southeastern Spain populations, respectively) and *m1* and *m2* = 0 (the migration rates between both populations). For scaling the demographic parameters to individuals and years, a generation time of 17.72 years was used, which corresponded to the mean estimates for the Doñana population^60^ and the mutation rates of loci (*μ*) were between 3 × 10^−9^ and 2 × 10^−7^. The mutation rates were calculated from the number of alleles at each locus and the divergence time of the *T. g. graeca* lineage^22^. Parameter convergence and chain mixing were assessed by ESS above 100, and the consistency of results across replicated runs.

### Ethic statements

Sampling of spur-thighed tortoises in Doñana was conducted under the authorization and following the protocols approved by the Junta de Andalucía (permit nos.: 21/2012 and 2013/15). The sampling protocols were approved by the Ethic Committee of the Miguel Hernández University (DBA-AGC-001-12), in accordance with the approved guidelines.

## Electronic supplementary material

Supplementary material
