# Supplementary material for: Human-mediated secondary contact of two tortoise lineages results in sex-biased introgression
Source: Sci Rep. 2017 Jun 22;7:4019. doi: 10.1038/s41598-017-04208-4 (PMC5481369; doi:10.1038/s41598-017-04208-4)
Supplement: Supplementary file 1 — Supplementary material [file 41598_2017_4208_MOESM1_ESM.pdf]

Supplementary material

Human-mediated secondary contact of two tortoise lineages results in sex-biased introgression

EVA GRACIÁ, ROBERTO C. RODRÍGUEZ-CARO, ANA C. ANDREU, UWE FRITZ, ANDRÉS GIMÉNEZ, FRANCISCO BOTELLA

Supplementary Table S1 Samples of western Mediterranean *Testudo graeca* and their haplotypes. genCODE refers to voucher samples in the collections of the Museum of Zoology Dresden (Germany; MZD), Doñana Biological Station (Spain; DBS), the Miguel Hernandez University Elche (Spain; MHU) or the Museum of Natural History of the University Florence (Italy; UF). The samples from Doñana were analyzed by first time, the remaining were available at GenBank from previous studies[1–5].

Subspecies	GenCODE	Collection	Locality	Cyt *b* haplotypes (GenBank accession number)
T. g. graeca	DO_1309	MHU-DBS	Doñana National Park: El Puntal	$B_1$35 (LT838800)
T. g. graeca	DO_1177	MHU-DBS	Doñana National Park: El Puntal	$B_1$35 (LT838800)
T. g. graeca	DO_1474	MHU-DBS	Doñana National Park: El Puntal	$B_1$35 (LT838801)
T. g. graeca	DO_1448	MHU-DBS	Doñana National Park: El Puntal	$B_1$35 (LT838800)
T. g. graeca	DO_1184	MHU-DBS	Doñana National Park: El Puntal	$B_1$35 (LT838800)
T. g. graeca	DO_1198	MHU-DBS	Doñana National Park: El Puntal	$B_1$35 (LT838800)
T. g. graeca	DO_1552	MHU-DBS	Doñana National Park: El Puntal	$B_1$35 (LT838800)
T. g. marokkensis	DO_1378	MHU-DBS	Doñana National Park: El Puntal	$B_2$1 (FM162032)
T. g. graeca	DO_1574	MHU-DBS	Doñana National Park: El Puntal	$B_1$35 (LT838801)
T. g. graeca	DO_1555	MHU-DBS	Doñana National Park: El Puntal	$B_1$35 (LT838800)
T. g. graeca	DO_1288	MHU-DBS	Doñana National Park: El Puntal	$B_1$35 (LT838801)
T. g. graeca	DO_1403	MHU-DBS	Doñana National Park: El Puntal	$B_1$35 (LT838800)
T. g. graeca	DO_1054	MHU-DBS	Doñana National Park: El Puntal	$B_1$35 (LT838801)
T. g. marokkensis	DO_1392	MHU-DBS	Doñana National Park: El Puntal	$B_2$1 (FM162032)
T. g. graeca	DO_1532	MHU-DBS	Doñana National Park: El Puntal	$B_1$35 (LT838800)
T. g. graeca	DO_1428	MHU-DBS	Doñana National Park: El Puntal	$B_1$35 (LT838800)
T. g. graeca	DO_1747	MHU	Doñana National Park: El Puntal	$B_1$35 (LT838800)
T. g. graeca	DO_1735	MHU	Doñana National Park: El Puntal	$B_1$35 (LT838801)
T. g. graeca	DO_4080	MHU	Doñana National Park: El Puntal	$B_1$35 (LT838800)
T. g. graeca	DO_4083	MHU	Doñana National Park: El Puntal	$B_1$35 (LT838800)
T. g. graeca	DO_654	MHU	Doñana National Park: El Puntal	$B_1$35 (LT838800)
T. g. marokkensis	DO_842	MHU	Doñana National Park: El Puntal	$B_2$1 (FM162032)
T. g. graeca	DO_1230	MHU	Doñana National Park: El Puntal	$B_1$35 (LT838800)
T. g. marokkensis	DO_1452	MHU	Doñana National Park: El Puntal	$B_2$1 (FM162032)
T. g. graeca	DO_4072	MHU	Doñana National Park: El Puntal	$B_1$35 (LT838800)
T. g. graeca	DO_1460	MHU	Doñana National Park: El Puntal	$B_1$35 (LT838800)
T. g. graeca	DO_4074	MHU	Doñana National Park: El Puntal	$B_1$35 (LT838800)
T. g. marokkensis	DO_1576	MHU	Doñana National Park: El Puntal	$B_2$1 (FM162032)
T. g. graeca	DO_1718	MHU	Doñana National Park: El Puntal	$B_1$35 (LT838801)
T. g. graeca	DO_2002	MHU	Doñana National Park: El Puntal	$B_1$35 (LT838800)
T. g. graeca	DO_1777	MHU	Doñana National Park: El Puntal	$B_1$35 (LT838800)
T. g. graeca	DO_1827	MHU	Doñana National Park: El Puntal	$B_1$35 (LT838800)
T. g. graeca	DO_4073	MHU	Doñana National Park: El Puntal	$B_1$35 (LT838801)
T. g. graeca	DO_4071	MHU	Doñana National Park: El Puntal	$B_1$35 (LT838800)
T. g. graeca	DO_4067	MHU	Doñana National Park: El Puntal	$B_1$35 (LT838801)
T. g. graeca	DO_4068	MHU	Doñana National Park: El Puntal	$B_1$35 (LT838800)
T. g. graeca	DO_731	MHU	Doñana National Park: El Puntal	$B_1$35 (LT838801)
T. g. graeca	DO_4095	MHU	Doñana National Park: El Puntal	$B_1$35 (LT838800)
T. g. graeca	DO_4077	MHU	Doñana National Park: El Puntal	$B_1$35 (LT838801)
T. g. graeca	DO_1487	MHU	Doñana National Park: El Puntal	$B_1$35 (LT838800)
T. g. graeca	DO_4078	MHU	Doñana National Park: El Puntal	$B_1$35 (LT838801)
T. g. graeca	DO_6732	MHU	Doñana National Park: El Puntal	$B_1$35 (LT838800)
T. g. graeca	DO_1811	MHU	Doñana National Park: El Puntal	$B_1$35 (LT838800)
T. g. graeca	DO_4089	MHU	Doñana National Park: El Puntal	$B_1$35 (LT838801)
T. g. graeca	DO_4101	MHU	Doñana National Park: El Puntal	$B_1$35 (LT838800)

T. g. graeca	DO_4096	MHU	Doñana National Park: El Puntal	$B_1$35 (LT838800)
T. g. graeca	DO_4085	MHU	Doñana National Park: El Puntal	$B_1$35 (LT838800)
T. g. graeca	DO_1056	MHU	Doñana National Park: El Puntal	$B_1$35 (LT838800)
T. g. graeca	DO_1674-5	MHU	Doñana National Park: El Puntal	$B_1$35 (LT838800)
T. g. graeca	DO_4093	MHU	Doñana National Park: El Puntal	$B_1$35 (LT838800)
T. g. marokkensis	DO_4084	MHU	Doñana National Park: El Puntal	$B_2$1 (FM162032)
T. g. graeca	DO_668	MHU	Doñana National Park: El Puntal	$B_1$35 (LT838800)
T. g. graeca	DO_832	MHU	Doñana National Park: El Puntal	$B_1$35 (LT838801)
T. g. graeca	DO_4075	MHU	Doñana National Park: El Puntal	$B_1$35 (LT838800)
T. g. graeca	DO_4102	MHU	Doñana National Park: El Puntal	$B_1$35 (LT838800)
T. g. graeca	DO_4097	MHU	Doñana National Park: El Puntal	$B_1$35 (LT838801)
T. g. graeca	DO_1167	MHU	Doñana National Park: El Puntal	$B_1$35 (LT838800)
T. g. graeca	DO_1665	MHU	Doñana National Park: El Puntal	$B_1$35 (LT838800)
T. g. graeca	DO_1444	MHU	Doñana National Park: El Puntal	$B_1$35 (LT838800)
T. g. graeca	DO_2007	MHU	Doñana National Park: El Puntal	$B_1$35 (LT838801)
T. g. marokkensis	DO_524	MHU	Doñana National Park: El Puntal	$B_2$1 (FM162032)
T. g. graeca	DO_4081	MHU	Doñana National Park: El Puntal	$B_1$35 (LT838800)
T. g. marokkensis	DO_917	MHU	Doñana National Park: El Puntal	$B_2$1 (FM162032)
T. g. graeca	DO_1401	MHU	Doñana National Park: El Puntal	$B_1$35 (LT838801)
T. g. graeca	DO_4094	MHU	Doñana National Park: El Puntal	$B_1$35 (LT838800)
T. g. graeca	DO_732	MHU	Doñana National Park: El Puntal	$B_1$35 (LT838800)
T. g. marokkensis	DO_4090	MHU	Doñana National Park: El Puntal	$B_2$1 (FM162032)
T. g. graeca	DO_4099	MHU	Doñana National Park: El Puntal	$B_1$35 (LT838800)
T. g. graeca	DO_4091	MHU	Doñana National Park: El Puntal	$B_1$35 (LT838800)
T. g. graeca	DO_4103	MHU	Doñana National Park: El Puntal	$B_1$35 (LT838800)
T. g. graeca	DO_6800	MHU	Doñana National Park: El Puntal	$B_1$35 (LT838800)
T. g. graeca	DO_1813	MHU	Doñana National Park: El Puntal	$B_1$35 (LT838801)
T. g. graeca	DO_4100	MHU	Doñana National Park: El Puntal	$B_1$35 (LT838801)
T. g. graeca	DO_586	MHU	Doñana National Park: El Puntal	$B_1$35 (LT838800)
T. g. graeca	DO_4079	MHU	Doñana National Park: El Puntal	$B_1$35 (LT838800)
T. g. graeca	DO_4082	MHU	Doñana National Park: El Puntal	$B_1$35 (LT838800)
T. g. graeca	DO_1725	MHU	Doñana National Park: El Puntal	$B_1$35 (LT838800)
T. g. graeca	DO_6755	MHU	Doñana National Park: El Puntal	$B_1$35 (LT838800)
T. g. graeca	DO_1074	MHU	Doñana National Park: El Puntal	$B_1$35 (LT838800)
T. g. graeca	DO_1629	MHU	Doñana National Park: El Puntal	$B_1$35 (LT838800)
T. g. graeca	DO_1822	MHU	Doñana National Park: El Puntal	$B_1$35 (LT838801)
T. g. graeca	DO2_4108	MHU	Doñana National Park: Marismillas	$B_1$35 (LT838800)
T. g. graeca	DO2_4105	MHU	Doñana National Park: Marismillas	$B_1$35 (LT838800)
T. g. graeca	DO2_4107	MHU	Doñana National Park: Marismillas	$B_1$35 (LT838800)
T. g. graeca	DO2_4106	MHU	Doñana National Park: Marismillas	$B_1$35 (LT838800)
T. g. graeca	AC01	MHU	Algeria: Ain Chorfa	$B_1$29 (HE585732)
T. g. graeca	AC02	MHU	Algeria: Ain Chorfa	$B_1$29 (HE585732)
T. g. graeca	AC03	MHU	Algeria: Ain Chorfa	$B_1$29 (HE585732)
T. g. graeca	AC04	MHU	Algeria: Ain Chorfa	$B_1$29 (HE585732)
T. g. graeca	AC05	MHU	Algeria: Ain Chorfa	$B_1$29 (HE585732)
T. g. graeca	AC06	MHU	Algeria: Ain Chorfa	$B_1$29 (HE585732)
T. g. graeca	AC07	MHU	Algeria: Ain Chorfa	$B_1$29 (HE585732)
T. g. graeca	AC08	MHU	Algeria: Ain Chorfa	$B_1$29 (HE585732)
T. g. graeca	AC09	MHU	Algeria: Ain Chorfa	$B_1$29 (HE585732)
T. g. graeca	AC10	MHU	Algeria: Ain Chorfa	$B_1$33 (HE585736)
T. g. graeca	AN01	MHU	Algeria: Ain Naga	$B_1$22 (HE585725)
T. g. graeca	AR01	MHU	Algeria: Algiers	$B_1$21 (HE585724)
T. g. graeca	4104	MZD	Algeria: Algiers	$B_1$3 (FM162028)
T. g. graeca	AM01	MHU	Algeria: Amoura	$B_1$2 (FM162027)
T. g. graeca	A1	MHU	Algeria: Annaba	$B_1$3 (FM162028)
T. g. graeca	4106	MZD	Algeria: Annaba	$B_1$3 (FM162028)
T. g. graeca	BI01	MHU	Algeria: Birkhadem	$B_1$3 (FM162028)
T. g. graeca	GA01	MHU	Algeria: Gare Aomar	$B_1$29 (HE585732)
T. g. graeca	G01	MHU	Algeria: Guelma	$B_1$3 (FM162028)
T. g. graeca	G03	MHU	Algeria: Guelma	$B_1$3 (FM162028)
T. g. graeca	G04	MHU	Algeria: Guelma	$B_1$3 (FM162028)
T. g. graeca	GU01	MHU	Algeria: Guertofa	$B_1$17 (HE585720)
T. g. graeca	GU05	MHU	Algeria: Guertofa	$B_1$17 (HE585720)
T. g. graeca	GU06	MHU	Algeria: Guertofa	$B_1$17 (HE585720)
T. g. graeca	GU08	MHU	Algeria: Guertofa	$B_1$17 (HE585720)
T. g. graeca	GU09	MHU	Algeria: Guertofa	$B_1$17 (HE585720)
T. g. graeca	GU03	MHU	Algeria: Guertofa	$B_1$19 (HE585722)
T. g. graeca	GU02	MHU	Algeria: Guertofa	$B_1$29 (HE585732)
T. g. graeca	GU04	MHU	Algeria: Guertofa	$B_1$29 (HE585732)
T. g. graeca	GU07	MHU	Algeria: Guertofa	$B_1$29 (HE585732)
T. g. graeca	GU11	MHU	Algeria: Guertofa	$B_1$29 (HE585732)
T. g. graeca	GU10	MHU	Algeria: Guertofa	$B_1$32 (HE585735)

T. g. graeca	KA01	MHU	Algeria: Kadiria	$B_1$3 (FM162028)
T. g. graeca	4101	MZD	Algeria: Khenchela	$B_1$3 (FM162028)
T. g. graeca	4102	MZD	Algeria: Khenchela	$B_1$3 (FM162028)
T. g. graeca	4103	MZD	Algeria: Khenchela	$B_1$3 (FM162028)
T. g. graeca	MS12	MHU	Algeria: Messad	$B_1$11 (HE585714)
T. g. graeca	MS05	MHU	Algeria: Messad	$B_1$11 (HE585714)
T. g. graeca	MS14	MHU	Algeria: Messad	$B_1$12 (HE585715)
T. g. graeca	MS13	MHU	Algeria: Messad	$B_1$14 (HE585717)
T. g. graeca	MS07	MHU	Algeria: Messad	$B_1$14 (HE585717)
T. g. graeca	MS06	MHU	Algeria: Messad	$B_1$29 (HE585732)
T. g. graeca	MS08	MHU	Algeria: Messad	$B_1$29 (HE585732)
T. g. graeca	MS11	MHU	Algeria: Messad	$B_1$29 (HE585732)
T. g. graeca	MS10	MHU	Algeria: Messad	$B_1$3 (FM162028)
T. g. graeca	MS09	MHU	Algeria: Messad	$B_1$31 (HE585734)
T. g. graeca	MB04	MHU	Algeria: Moudjbara	$B_1$11 (HE585714)
T. g. graeca	MB10	MHU	Algeria: Moudjbara	$B_1$13 (HE585716)
T. g. graeca	MB01	MHU	Algeria: Moudjbara	$B_1$13 (HE585716)
T. g. graeca	MB02	MHU	Algeria: Moudjbara	$B_1$13 (HE585716)
T. g. graeca	MB14	MHU	Algeria: Moudjbara	$B_1$23 (HE585726)
T. g. graeca	MB06	MHU	Algeria: Moudjbara	$B_1$23 (HE585726)
T. g. graeca	MB08	MHU	Algeria: Moudjbara	$B_1$23 (HE585726)
T. g. graeca	MB15	MHU	Algeria: Moudjbara	$B_1$23 (HE585726)
T. g. graeca	MB00	MHU	Algeria: Moudjbara	$B_1$23 (HE585726)
T. g. graeca	MB05	MHU	Algeria: Moudjbara	$B_1$23 (HE585726)
T. g. graeca	MB07	MHU	Algeria: Moudjbara	$B_1$23 (HE585726)
T. g. graeca	MB11	MHU	Algeria: Moudjbara	$B_1$23 (HE585726)
T. g. graeca	MB16	MHU	Algeria: Moudjbara	$B_1$23 (HE585726)
T. g. graeca	MB17	MHU	Algeria: Moudjbara	$B_1$23 (HE585726)
T. g. graeca	5391	MZD	Algeria: Mountains near Tiaret (confiscated)	$B_1$29 (HE585732)
T. g. graeca	5396	MZD	Algeria: Mountains near Tiaret (confiscated)	$B_1$29 (HE585732)
T. g. graeca	5144	MZD	Algeria: Oran	$B_1$3 (FM162028)
T. g. graeca	SS01	MHU	Algeria: Saf Saf	$B_1$2 (FM162027)
T. g. graeca	SS02	MHU	Algeria: Saf Saf	$B_1$2 (FM162027)
T. g. graeca	SS03	MHU	Algeria: Saf Saf	$B_1$2 (FM162027)
T. g. graeca	SS05	MHU	Algeria: Saf Saf	$B_1$2 (FM162027)
T. g. graeca	SS09	MHU	Algeria: Saf Saf	$B_1$2 (FM162027)
T. g. graeca	SS07	MHU	Algeria: Saf Saf	$B_1$26 (HE585729)
T. g. graeca	SS04	MHU	Algeria: Saf Saf	$B_1$27 (HE585730)
T. g. graeca	SS06	MHU	Algeria: Saf Saf	$B_1$28 (HE585731)
T. g. graeca	SS10	MHU	Algeria: Saf Saf	$B_1$29 (HE585732)
T. g. graeca	SS11	MHU	Algeria: Saf Saf	$B_1$29 (HE585732)
T. g. graeca	SS08	MHU	Algeria: Saf Saf	$B_1$8 (HE588137)
T. g. graeca	SB02	MHU	Algeria: Sidi M'Hamed Benaouda	$B_1$2 (FM162027)
T. g. graeca	SB01	MHU	Algeria: Sidi M'Hamed Benaouda	$B_1$30 (HE585733)
T. g. graeca	TH05	MHU	Algeria: Theniet el Had	$B_1$24 (HE585727)
T. g. graeca	TH01	MHU	Algeria: Theniet el Had	$B_1$3 (FM162028)
T. g. graeca	TH02	MHU	Algeria: Theniet el Had	$B_1$3 (FM162028)
T. g. graeca	TH03	MHU	Algeria: Theniet el Had	$B_1$3 (FM162028)
T. g. graeca	TH06	MHU	Algeria: Theniet el Had	$B_1$3 (FM162028)
T. g. graeca	TH07	MHU	Algeria: Theniet el Had	$B_1$3 (FM162028)
T. g. graeca	KH03	MHU	Algeria: Zemmora	$B_1$18 (HE585721)
T. g. graeca	KH02	MHU	Algeria: Zemmora	$B_1$2 (FM162027)
T. g. graeca	KH04	MHU	Algeria: Zemmora	$B_1$20 (HE585723)
T. g. graeca	KH01	MHU	Algeria: Zemmora	$B_1$29 (HE585732)
T. g. graeca	ZE01	MHU	Algeria: Zemouri-Leguata	$B_1$25 (HE585728)
T. g. graeca	ZE02	MHU	Algeria: Zemouri-Leguata	$B_1$3 (FM162028)
T. g. graeca	ZE03	MHU	Algeria: Zemouri-Leguata	$B_1$3 (FM162028)
T. g. graeca	ZE04	MHU	Algeria: Zemouri-Leguata	$B_1$3 (FM162028)
T. g. graeca	ZE06	MHU	Algeria: Zemouri-Leguata	$B_1$3 (FM162028)
T. g. graeca	ZE07	MHU	Algeria: Zemouri-Leguata	$B_1$3 (FM162028)
T. g. graeca	ZE08	MHU	Algeria: Zemouri-Leguata	$B_1$3 (FM162028)
T. g. graeca	ZE09	MHU	Algeria: Zemouri-Leguata	$B_1$3 (FM162028)
T. g. graeca	ZE10	MHU	Algeria: Zemouri-Leguata	$B_1$3 (FM162028)
T. g. graeca	42819	MZD	Libya: Cyrenaica	$B_1$3 (FM162028)
T. g. graeca	D 42822	MZD	Spain: Majorca (Calvià Nord)	$B_1$6 (FM162031)
T. g. graeca	MALL-01	MHU	Spain: Majorca (Cala Figuera)	$B_1$2 (FM162027)
T. g. graeca	MALL-02	MHU	Spain: Majorca (Cala Figuera)	$B_1$2 (FM162027)
T. g. graeca	MALL-03	MHU	Spain: Majorca (Cala Figuera)	$B_1$2 (FM162027)
T. g. graeca	MALL-04	MHU	Spain: Majorca (Calvià Nord)	$B_1$2 (FM162027)
T. g. graeca	MALL-05	MHU	Spain: Majorca (Calvià Nord)	$B_1$2 (FM162027)
T. g. graeca	MALL-06	MHU	Spain: Majorca (Calvià Nord)	$B_1$2 (FM162027)
T. g. graeca	MALL-07	MHU	Spain: Majorca (confiscated)	$B_1$2 (FM162027)

T. g. graeca	MALL-08	MHU	Spain: Majorca (confiscated)	$B_1$2 (FM162027)
T. g. graeca	MALL-09	MHU	Spain: Majorca (confiscated)	$B_1$2 (FM162027)
T. g. graeca	MALL-10	MHU	Spain: Majorca (confiscated)	$B_1$2 (FM162027)
T. g. graeca	MALL-11	MHU	Spain: Majorca (confiscated)	$B_1$2 (FM162027)
T. g. graeca	4444	MZD	Morocco: Central Jbilet Mountains	$B_1$3 (FM162028)
T. g. graeca	4451	MZD	Morocco: Debdou	$B_1$5 (FM162030)
T. g. graeca	MO02	MHU	Morocco: Moulouya	$B_1$10 (HE585713)
T. g. graeca	MO07	MHU	Morocco: Moulouya	$B_1$34 (HE585737)
T. g. graeca	MO04	MHU	Morocco: Moulouya	$B_1$6 (FM162031)
T. g. graeca	MO05	MHU	Morocco: Moulouya	$B_1$6 (FM162031)
T. g. graeca	MO06	MHU	Morocco: Moulouya	$B_1$6 (FM162031)
T. g. graeca	MO03	MHU	Morocco: Moulouya	$B_1$9 (HE588138)
T. g. graeca	4447	MZD	Morocco: Moulouya River Mouth	$B_1$6 (FM162031)
T. g. graeca	4448	MZD	Morocco: Moulouya River Mouth	$B_1$6 (FM162031)
T. g. graeca	2294	MZD	Morocco: NE Otat Oulad el Hajj	$B_1$7 (AM230965)
T. g. graeca	KA11	MHU	Morocco: Sidi Redouane	$B_1$6 (FM162031)
T. g. graeca	5187	MZD-MHU	Spain: Bas Sur	$B_1$1 (FM162026)
T. g. graeca	5188	MZD-MHU	Spain: Bas Sur	$B_1$1 (FM162026)
T. g. graeca	5189	MZD-MHU	Spain: Bas Sur	$B_1$1 (FM162026)
T. g. graeca	5190	MZD-MHU	Spain: Bas Sur	$B_1$1 (FM162026)
T. g. graeca	5191	MZD-MHU	Spain: Bas Sur	$B_1$3 (FM162028)
T. g. graeca	5207	MZD-MHU	Spain: Centinares	$B_1$4 (FM162029)
T. g. graeca	5208	MZD-MHU	Spain: Centinares	$B_1$4 (FM162029)
T. g. graeca	5209	MZD-MHU	Spain: Centinares	$B_1$4 (FM162029)
T. g. graeca	5210	MZD-MHU	Spain: Centinares	$B_1$4 (FM162029)
T. g. graeca	5211	MZD-MHU	Spain: Centinares	$B_1$4 (FM162029)
T. g. graeca	ZE02	MHU	Spain: Cerrichera	$B_1$1 (FM162026)
T. g. graeca	ZE05	MHU	Spain: Cerrichera	$B_1$1 (FM162026)
T. g. graeca	ZE07	MHU	Spain: Cerrichera	$B_1$1 (FM162026)
T. g. graeca	ZE09	MHU	Spain: Cerrichera	$B_1$1 (FM162026)
T. g. graeca	ZE11	MHU	Spain: Cerrichera	$B_1$1 (FM162026)
T. g. graeca	ZE01	MHU	Spain: Cerrichera	$B_1$3 (FM162028)
T. g. graeca	ZE03	MHU	Spain: Cerrichera	$B_1$3 (FM162028)
T. g. graeca	ZE06	MHU	Spain: Cerrichera	$B_1$3 (FM162028)
T. g. graeca	ZE08	MHU	Spain: Cerrichera	$B_1$3 (FM162028)
T. g. graeca	CH21	MHU	Spain: Chinas	$B_1$1 (FM162026)
T. g. graeca	CH09	MHU	Spain: Chinas	$B_1$1 (FM162026)
T. g. graeca	CH11	MHU	Spain: Chinas	$B_1$4 (FM162029)
T. g. graeca	CH10	MHU	Spain: Chinas	$B_1$4 (FM162029)
T. g. graeca	CH19	MHU	Spain: Chinas	$B_1$4 (FM162029)
T. g. graeca	CH12	MHU	Spain: Chinas	$B_1$4 (FM162029)
T. g. graeca	CH15	MHU	Spain: Chinas	$B_1$4 (FM162029)
T. g. graeca	CH17	MHU	Spain: Chinas	$B_1$4 (FM162029)
T. g. graeca	CH20	MHU	Spain: Chinas	$B_1$4 (FM162029)
T. g. graeca	CH22	MHU	Spain: Chinas	$B_1$4 (FM162029)
T. g. graeca	CR09	MHU	Spain: Crisoleja	$B_1$1 (FM162026)
T. g. graeca	CR11	MHU	Spain: Crisoleja	$B_1$1 (FM162026)
T. g. graeca	CR01	MHU	Spain: Crisoleja	$B_1$3 (FM162028)
T. g. graeca	CR02	MHU	Spain: Crisoleja	$B_1$3 (FM162028)
T. g. graeca	CR03	MHU	Spain: Crisoleja	$B_1$3 (FM162028)
T. g. graeca	CR05	MHU	Spain: Crisoleja	$B_1$3 (FM162028)
T. g. graeca	CR07	MHU	Spain: Crisoleja	$B_1$3 (FM162028)
T. g. graeca	CR08	MHU	Spain: Crisoleja	$B_1$3 (FM162028)
T. g. graeca	CR10	MHU	Spain: Crisoleja	$B_1$3 (FM162028)
T. g. graeca	CU03	MHU	Spain: Culebras	$B_1$1 (FM162026)
T. g. graeca	CU04	MHU	Spain: Culebras	$B_1$1 (FM162026)
T. g. graeca	CU06	MHU	Spain: Culebras	$B_1$16 (HE585719)
T. g. graeca	CU02	MHU	Spain: Culebras	$B_1$3 (FM162028)
T. g. graeca	CU05	MHU	Spain: Culebras	$B_1$3 (FM162028)
T. g. graeca	CU07	MHU	Spain: Culebras	$B_1$3 (FM162028)
T. g. graeca	CU08	MHU	Spain: Culebras	$B_1$3 (FM162028)
T. g. graeca	CU09	MHU	Spain: Culebras	$B_1$3 (FM162028)
T. g. graeca	CU10	MHU	Spain: Culebras	$B_1$3 (FM162028)
T. g. graeca	CU11	MHU	Spain: Culebras	$B_1$3 (FM162028)
T. g. graeca	5192	MZD-MHU	Spain: Galera	$B_1$1 (FM162026)
T. g. graeca	5193	MZD-MHU	Spain: Galera	$B_1$1 (FM162026)
T. g. graeca	5195	MZD-MHU	Spain: Galera	$B_1$1 (FM162026)
T. g. graeca	5196	MZD-MHU	Spain: Galera	$B_1$1 (FM162026)
T. g. graeca	5194	MZD-MHU	Spain: Galera	$B_1$3 (FM162028)
T. g. graeca	JU03	MHU	Spain: Judio	$B_1$4 (FM162029)
T. g. graeca	JU06	MHU	Spain: Judio	$B_1$4 (FM162029)
T. g. graeca	JU07	MHU	Spain: Judio	$B_1$4 (FM162029)

T. g. graeca	JU08	MHU	Spain: Judio	$B_1$4 (FM162029)
T. g. graeca	JU09	MHU	Spain: Judio	$B_1$4 (FM162029)
T. g. graeca	LU05	MHU	Spain: Luchena	$B_1$1 (FM162026)
T. g. graeca	LU08	MHU	Spain: Luchena	$B_1$1 (FM162026)
T. g. graeca	LU12	MHU	Spain: Luchena	$B_1$1 (FM162026)
T. g. graeca	LU02	MHU	Spain: Luchena	$B_1$3 (FM162028)
T. g. graeca	LU03	MHU	Spain: Luchena	$B_1$3 (FM162028)
T. g. graeca	LU04	MHU	Spain: Luchena	$B_1$3 (FM162028)
T. g. graeca	LU06	MHU	Spain: Luchena	$B_1$3 (FM162028)
T. g. graeca	LU07	MHU	Spain: Luchena	$B_1$3 (FM162028)
T. g. graeca	LU10	MHU	Spain: Luchena	$B_1$3 (FM162028)
T. g. graeca	5202	MZD-MHU	Spain: Malacate	$B_1$1 (FM162026)
T. g. graeca	5203	MZD-MHU	Spain: Malacate	$B_1$1 (FM162026)
T. g. graeca	5204	MZD-MHU	Spain: Malacate	$B_1$1 (FM162026)
T. g. graeca	5205	MZD-MHU	Spain: Malacate	$B_1$1 (FM162026)
T. g. graeca	5206	MZD-MHU	Spain: Malacate	$B_1$1 (FM162026)
T. g. graeca	MA03	MHU	Spain: Marinica	$B_1$15 (HE585718)
T. g. graeca	MA09	MHU	Spain: Marinica	$B_1$15 (HE585718)
T. g. graeca	MA04	MHU	Spain: Marinica	$B_1$16 (HE585719)
T. g. graeca	MA01	MHU	Spain: Marinica	$B_1$16 (HE585719)
T. g. graeca	MA05	MHU	Spain: Marinica	$B_1$16 (HE585719)
T. g. graeca	MA06	MHU	Spain: Marinica	$B_1$16 (HE585719)
T. g. graeca	MA07	MHU	Spain: Marinica	$B_1$16 (HE585719)
T. g. graeca	MA08	MHU	Spain: Marinica	$B_1$16 (HE585719)
T. g. graeca	MA10	MHU	Spain: Marinica	$B_1$16 (HE585719)
T. g. graeca	MA11	MHU	Spain: Marinica	$B_1$2 (FM162027)
T. g. graeca	5198	MZD-MHU	Spain: Palas	$B_1$1 (FM162026)
T. g. graeca	5199	MZD-MHU	Spain: Palas	$B_1$1 (FM162026)
T. g. graeca	5200	MZD-MHU	Spain: Palas	$B_1$1 (FM162026)
T. g. graeca	5201	MZD-MHU	Spain: Palas	$B_1$1 (FM162026)
T. g. graeca	5197	MZD-MHU	Spain: Palas	$B_1$3 (FM162028)
T. g. graeca	SO09	MHU	Spain: Sotomayor	$B_1$1 (FM162026)
T. g. graeca	SO10	MHU	Spain: Sotomayor	$B_1$1 (FM162026)
T. g. graeca	SO12	MHU	Spain: Sotomayor	$B_1$1 (FM162026)
T. g. graeca	SO13	MHU	Spain: Sotomayor	$B_1$1 (FM162026)
T. g. graeca	SO15	MHU	Spain: Sotomayor	$B_1$1 (FM162026)
T. g. graeca	SO16	MHU	Spain: Sotomayor	$B_1$1 (FM162026)
T. g. graeca	SO17	MHU	Spain: Sotomayor	$B_1$1 (FM162026)
T. g. graeca	SO18	MHU	Spain: Sotomayor	$B_1$1 (FM162026)
T. g. graeca	SO19	MHU	Spain: Sotomayor	$B_1$1 (FM162026)
T. g. graeca	46394	MZD	Spain: South-eastern Spain (captive animal)	$B_1$2 (FM162027)
T. g. marokkensis	5468	MZD	Morocco: Ain Bouali (Fès)	$B_2$1 (FM162032)
T. g. marokkensis	5471	MZD	Morocco: Ain Bouali (Fès)	$B_2$1 (FM162032)
T. g. marokkensis	5472	MZD	Morocco: Ain Bouali (Fès)	$B_2$1 (FM162032)
T. g. marokkensis	5445	MZD	Morocco: Ain Bouali (Fès)	$B_2$2 (HE585738)
T. g. marokkensis	5446	MZD	Morocco: Ain Bouali (Fès)	$B_2$2 (HE585738)
T. g. marokkensis	5469	MZD	Morocco: Ain Bouali (Fès)	$B_2$3 (HE585739)
T. g. marokkensis	5470	MZD	Morocco: Ain Bouali (Fès)	$B_2$4 (HE585740)
T. g. marokkensis	5235	MZD	Morocco: Arbaoua, near the barrage of Al Makhazine	$B_2$1 (FM162032)
T. g. marokkensis	5236	MZD	Morocco: Arbaoua, near the barrage of Al Makhazine	$B_2$1 (FM162032)
T. g. marokkensis	4446	MZD	Morocco: Balcon D'Ito	$B_2$1 (FM162032)
T. g. marokkensis	EH1	MHU	Morocco: Harcha-Oulmés	$B_2$1 (FM162032)
T. g. marokkensis	EH2b	MHU	Morocco: Harcha-Oulmés	$B_2$1 (FM162032)
T. g. marokkensis	EH3	MHU	Morocco: Harcha-Oulmés	$B_2$1 (FM162032)
T. g. marokkensis	EH2a	MHU	Morocco: Harcha-Oulmés	$B_2$7 (HE585743)
T. g. marokkensis	KB1	MHU	Morocco: Kariat Ba Mohammed	$B_2$1 (FM162032)
T. g. marokkensis	KB2	MHU	Morocco: Kariat Ba Mohammed	$B_2$1 (FM162032)
T. g. marokkensis	5237	MZD	Morocco: Near Lalla Mimouna	$B_2$1 (FM162032)
T. g. marokkensis	SA1	MHU	Morocco: Near Volubilis, Moulay Driss Zerhoune	$B_2$1 (FM162032)
T. g. marokkensis	SA2	MHU	Morocco: Near Volubilis, Moulay Driss Zerhoune	$B_2$1 (FM162032)
T. g. marokkensis	SA3	MHU	Morocco: Near Volubilis, Moulay Driss Zerhoune	$B_2$1 (FM162032)
T. g. marokkensis	4182	MZD	Morocco: Oudrass, S Tétouan	$B_2$1 (FM162032)
T. g. marokkensis	4183	MZD	Morocco: Oudrass, S Tétouan	$B_2$1 (FM162032)
T. g. marokkensis	4184	MZD	Morocco: Oudrass, S Tétouan	$B_2$1 (FM162032)
T. g. marokkensis	5366	MZD	Morocco: Oued Errif, Douar El Bacha	$B_2$1 (FM162032)
T. g. marokkensis	OU1	MHU	Morocco: Oued Khémis	$B_2$1 (FM162032)
T. g. marokkensis	OU2	MHU	Morocco: Oued Khémis	$B_2$1 (FM162032)

T. g. marokkensis	RH1	MHU	Morocco: Oued Miet. Between Béni Ammar and Moulay Driss Zerhoune	$B_2$1 (FM162032)
T. g. marokkensis	5425	MZD	Morocco: Oulmès	$B_2$1 (FM162032)
T. g. marokkensis	5426	MZD	Morocco: Oulmès	$B_2$1 (FM162032)
T. g. marokkensis	5424	MZD	Morocco: Oulmès	$B_2$5 (HE585741)
T. g. marokkensis	5423	MZD	Morocco: Oulmès	$B_2$5 (HE585741)
T. g. marokkensis	5450	MZD	Morocco: Oulmèss	$B_2$5 (HE585741)
T. g. marokkensis	5451	MZD	Morocco: Oulmes	$B_2$5 (HE585741)
T. g. marokkensis	5453	MZD	Morocco: Oulmes	$B_2$5 (HE585741)
T. g. marokkensis	5455	MZD	Morocco: Oulmes	$B_2$5 (HE585741)
T. g. marokkensis	5456	MZD	Morocco: Oulmes	$B_2$5 (HE585741)
T. g. marokkensis	5452	MZD	Morocco: Oulmes	$B_2$9 (HE585745)
T. g. marokkensis	5454	MZD	Morocco: Oulmes	$B_2$9 (HE585745)
T. g. marokkensis	KA01	MHU	Morocco: Sidi Redouane	$B_2$1 (FM162032)
T. g. marokkensis	KA02	MHU	Morocco: Sidi Redouane	$B_2$1 (FM162032)
T. g. marokkensis	KA04	MHU	Morocco: Sidi Redouane	$B_2$1 (FM162032)
T. g. marokkensis	KA06	MHU	Morocco: Sidi Redouane	$B_2$1 (FM162032)
T. g. marokkensis	KA07	MHU	Morocco: Sidi Redouane	$B_2$1 (FM162032)
T. g. marokkensis	KA09	MHU	Morocco: Sidi Redouane	$B_2$1 (FM162032)
T. g. marokkensis	KA10	MHU	Morocco: Sidi Redouane	$B_2$1 (FM162032)
T. g. marokkensis	KA12	MHU	Morocco: Sidi Redouane	$B_2$8 (HE585744)
T. g. marokkensis	KA05	MHU	Morocco: Sidi Redouane	$B_2$8 (HE585744)
T. g. marokkensis	SM	MHU	Morocco: Smir	$B_2$1 (FM162032)
T. g. marokkensis	SL1b	MHU	Morocco: Souk Letnin	$B_2$1 (FM162032)
T. g. marokkensis	SL1a	MHU	Morocco: Souk Letnin	$B_2$9 (HE585745)
T. g. marokkensis	5457	MZD	Morocco: Souss Valley: Admine Forest	$B_2$1 (FM162032)
T. g. marokkensis	JK1	MHU	Morocco: south Bab Taza	$B_2$6 (HE585742)
T. g. nabeulensis	E1	MHU	Algeria: El Kala	A6 (FM162023)
T. g. nabeulensis	E2	MHU	Algeria: El Kala	A6 (FM162023)
T. g. nabeulensis	E5	MHU	Algeria: El Kala	A6 (FM162023)
T. g. nabeulensis	E6	MHU	Algeria: El Kala	A6 (FM162023)
T. g. nabeulensis	E7	MHU	Algeria: El Kala	A6 (FM162023)
T. g. nabeulensis	E8	MHU	Algeria: El Kala	A6 (FM162023)
T. g. nabeulensis	E9	MHU	Algeria: El Kala	A6 (FM162023)
T. g. nabeulensis	E4	MHU	Algeria: El Kala	A7 (FM162024)
T. g. nabeulensis	3250	MZD	Algeria: El Kala	A7 (FM162024)
T. g. nabeulensis	E3	MHU	Algeria: El Kala	A7 (FM162024)
T. g. nabeulensis	5145	MZD	Algeria: Skikda	A6 (FM162023)
T. g. nabeulensis	930	MZD	Confiscated	A2 (FM162020)
T. g. nabeulensis	1899	MZD	Confiscated	A3 (FM162021)
T. g. nabeulensis	6460	MZD	Libya: unknown exact origin	A11 (HE585711)
T. g. nabeulensis	6451	MZD	Libya: unknown exact origin	A12 (HE585712)
T. g. nabeulensis	6453	MZD	Libya: unknown exact origin	A2 (FM162020)
T. g. nabeulensis	6456	MZD	Libya: unknown exact origin	A2 (FM162020)
T. g. nabeulensis	6459	MZD	Libya: unknown exact origin	A2 (FM162020)
T. g. nabeulensis	6454	MZD	Libya: unknown exact origin	A4 (FM162022)
T. g. nabeulensis	6455	MZD	Libya: unknown exact origin	A4 (FM162022)
T. g. nabeulensis	6458	MZD	Libya: unknown exact origin	A4 (FM162022)
T. g. nabeulensis	5703	MZD	Sardinia: Mal di Ventre Island	A10 (FR686466)
T. g. nabeulensis	5700	MZD	Sardinia: Mal di Ventre Island	A6 (FM162023)
T. g. nabeulensis	5701	MZD	Sardinia: Mal di Ventre Island	A6 (FM162023)
T. g. nabeulensis	5702	MZD	Sardinia: Mal di Ventre Island	A6 (FM162023)
T. g. nabeulensis	5704	MZD	Sardinia: Mal di Ventre Island	A6 (FM162023)
T. g. nabeulensis	5705	MZD	Sardinia: Mal di Ventre Island	A6 (FM162023)
T. g. nabeulensis	5706	MZD	Sardinia: Mal di Ventre Island	A6 (FM162023)
T. g. nabeulensis	5707	MZD	Sardinia: Mal di Ventre Island	A6 (FM162023)
T. g. nabeulensis	5710	MZD	Sardinia: Mal di Ventre Island	A6 (FM162023)
T. g. nabeulensis	CER-01	MHU	Sardinia: Mari Ermi	A6 (FM162023)
T. g. nabeulensis	CER-02	MHU	Sardinia: Mari Ermi	A6 (FM162023)
T. g. nabeulensis	CER-03	MHU	Sardinia: Mari Ermi	A6 (FM162023)
T. g. nabeulensis	CER-04	MHU	Sardinia: Mari Ermi	A6 (FM162023)
T. g. nabeulensis	CER-05	MHU	Sardinia: Mari Ermi	A6 (FM162023)
T. g. nabeulensis	5695	MZD	Sardinia: Narbolia, Montiferru	A6 (FM162023)
T. g. nabeulensis	5708	MZD	Sardinia: Narbolia, Montiferru	A6 (FM162023)
T. g. nabeulensis	5709	MZD	Sardinia: Narbolia, Montiferru	A6 (FM162023)
T. g. nabeulensis	NA2	UF-MHU	Sardinia: Narbolia, Montiferru	A6 (FM162023)
T. g. nabeulensis	1113	MZD	Sardinia: Putzu Idu	A6 (FM162023)
T. g. nabeulensis	5698	MZD	Sardinia: Putzu Idu	A6 (FM162023)
T. g. nabeulensis	5699	MZD	Sardinia: Seu	A6 (FM162023)
T. g. nabeulensis	5696	MZD	Sardinia: Seu	A6 (FM162023)
T. g. nabeulensis	5697	MZD	Sardinia: Seu	A6 (FM162023)
T. g. nabeulensis	SEU2	UF-MHU	Sardinia: Seu	A6 (FM162023)

T. g. nabeulensis	SEU3	UF-MHU	Sardinia: Seu	A6 (FM162023)
T. g. nabeulensis	SEU4	UF-MHU	Sardinia: Seu	A6 (FM162023)
T. g. nabeulensis	5044	MZD	Sardinia: Sinis Peninsula	A6 (FM162023)
T. g. nabeulensis	5045	MZD	Sardinia: Sinis Peninsula	A6 (FM162023)
T. g. nabeulensis	1112	MZD	Sardinia: Sinis Peninsula: 5 km NW S. Giovanni di Sinis	A6 (FM162023)
T. g. nabeulensis	PZ	UF-MHU	Sardinia: Torre del Pozo	A6 (FM162023)
T. g. nabeulensis	SE	UF-MHU	Sardinia: Seneghe	A6 (FM162023)
T. g. nabeulensis	2230	MZD	Sicily: Marsala	A2 (FM162020)
T. g. nabeulensis	4151	MZD	Tunisia: Djebel Bou Kornine near Hamam Lif	A2 (FM162020)
T. g. nabeulensis	800	MZD	Tunisia: Nabeul	A4 (FM162022)
T. g. nabeulensis	42883	MZD	Tunisia (confiscated)	A2 (FM162020)
T. g. nabeulensis	44865	MZD	Tunisia (confiscated)	A2 (FM162020)
T. g. nabeulensis	46397	MZD	Tunisia (confiscated)	A3 (FM162021)
T. g. nabeulensis	41852	MZD	Tunisia (confiscated)	A4 (FM162022)
T. g. nabeulensis	42893	MZD	Tunisia (confiscated)	A4 (FM162022)
T. g. nabeulensis	44864	MZD	Tunisia (confiscated)	A4 (FM162022)
T. g. nabeulensis	44857	MZD	Tunisia (confiscated)	A6 (FM162023)
T. g. nabeulensis	46588	MZD	Tunisia (confiscated)	A6 (FM162023)
T. g. nabeulensis	149	MZD	Tunisia: Sousse	A1 (AM230971)
T. g. nabeulensis	5002	MZD	Tunisia: Tabarka	A6 (FM162023)
T. g. nabeulensis	5003	MZD	Tunisia: Tabarka	A6 (FM162023)
T. g. nabeulensis	5005	MZD	Tunisia: Tabarka	A6 (FM162023)
T. g. nabeulensis	5004	MZD	Tunisia: Tabarka	A8 (FM162025)
T. g. soussensis	3826	MZD	Morocco: Aït-Ourir	D6 (FM162040)
T. g. soussensis	3818	MZD	Morocco: approx. 25 Km N Marrakech	D1 (FM162035)
T. g. soussensis	3817	MZD	Morocco: approx. 25 Km N Marrakech	D3 (FM162037)
T. g. soussensis	4442	MZD	Morocco: Central Jbilet Mountains	D1 (FM162035)
T. g. soussensis	4441	MZD	Morocco: Central Jbilet Mountains	D6 (FM162040)
T. g. soussensis	4443	MZD	Morocco: Central Jbilet Mountains	D6 (FM162040)
T. g. soussensis	4445	MZD	Morocco: Central Jbilet Mountains	D6 (FM162040)
T. g. soussensis	4449	MZD	Morocco: Demnate region: Iminifri	D5 (FM162039)
T. g. soussensis	4450	MZD	Morocco: Demnate region: Iminifri	D5 (FM162039)
T. g. soussensis	4431	MZD	Morocco: Essaouira	D4 (FM162038)
T. g. soussensis	4432	MZD	Morocco: Essaouira	D4 (FM162038)
T. g. soussensis	4433	MZD	Morocco: Essaouira	D4 (FM162038)
T. g. soussensis	4434	MZD	Morocco: Essaouira	D4 (FM162038)
T. g. soussensis	4435	MZD	Morocco: Essaouira	D9 (FM162043)
T. g. soussensis	3815	MZD	Morocco: Foothills of Jbel Amsitene	D3 (FM162037)
T. g. soussensis	5272	MZD	Morocco: near Rabat	D11 (HE585747)
T. g. soussensis	5036	MZD	Morocco: Sidi Toual	D8 (FM162042)
T. g. soussensis	5433	MZD	Morocco: Souss Valley, Admine Forest	D2 (FM162036)
T. g. soussensis	4439	MZD	Morocco: Souss Valley, Admine Forest	D2 (FM162036)
T. g. soussensis	5459	MZD	Morocco: Souss Valley, Admine Forest	D2 (FM162036)
T. g. soussensis	5432	MZD	Morocco: Souss Valley, Admine Forest	D3 (FM162037)
T. g. soussensis	4436	MZD	Morocco: Souss Valley, Admine Forest	D4 (FM162038)
T. g. soussensis	5430	MZD	Morocco: Souss Valley, Admine Forest	D8 (FM162042)
T. g. soussensis	4438	MZD	Morocco: Souss Valley, Admine Forest	D8 (FM162042)
T. g. soussensis	4440	MZD	Morocco: Souss Valley, Admine Forest	D8 (FM162042)
T. g. soussensis	5431	MZD	Morocco: Souss Valley, Admine Forest	D8 (FM162042)
T. g. soussensis	5458	MZD	Morocco: Souss Valley, Admine Forest	D8 (FM162042)
T. g. soussensis	5460	MZD	Morocco: Souss Valley, Admine Forest	D8 (FM162042)
T. g. soussensis	4437	MZD	Morocco: Souss Valley, Admine Forest	D9 (FM162043)
T. g. soussensis	5429	MZD	Morocco: Souss Valley, Admine Forest	D9 (FM162043)
T. g. soussensis	4426	MZD	Morocco: Tafrayate	D3 (FM162037)
T. g. soussensis	4427	MZD	Morocco: Tafrayate	D3 (FM162037)
T. g. soussensis	4429	MZD	Morocco: Tafrayate	D3 (FM162037)
T. g. soussensis	4430	MZD	Morocco: Tafrayate	D6 (FM162040)
T. g. soussensis	4428	MZD	Morocco: Tafrayate	D7 (FM162041)
T. g. soussensis	5441	MZD	Morocco: Taroudant	D10 (HE585746)
T. g. soussensis	5438	MZD	Morocco: Taroudant	D2 (FM162036)
T. g. soussensis	5440	MZD	Morocco: Taroudannt	D2 (FM162036)
T. g. soussensis	5437	MZD	Morocco: Taroudannt	D3 (FM162037)
T. g. soussensis	5442	MZD	Morocco: Taroudannt	D3 (FM162037)
T. g. soussensis	5461	MZD	Morocco: Taroudannt	D3 (FM162037)
T. g. soussensis	5439	MZD	Morocco: Taroudannt	D8 (FM162042)
T. g. soussensis	5443	MZD	Morocco: Taroudannt	D8 (FM162042)
T. g. soussensis	5436	MZD	Morocco: Taroudannt	D9 (FM162043)
T. g. soussensis	5462	MZD	Morocco: Tnine d'Ourika	D3 (FM162037)
T. g. soussensis	5463	MZD	Morocco: Tnine de l'Ourika	D3 (FM162037)
T. g. soussensis	5464	MZD	Morocco: Tnine de l'Ourika	D3 (FM162037)

T. g. soussensis	5465	MZD	Morocco: Tnine de l'Ourika	D3 (FM162037)
T. g. soussensis	5466	MZD	Morocco: Tnine de l'Ourika	D3 (FM162037)
T. g. cyrenaica	4496	MZD	Libya: Cyrenaica: Al-Kouf	C1(FM162033)
T. g. cyrenaica	5046	MZD	Libya: Slonta	C2 (FM162034)
T. g. cyrenaica	5047	MZD	Libya: Taknis	C2(FM162034)
T. g. cyrenaica	6450	MZD	Libya: unknown exact origin	C1 (FM162033)
**Unnamed lineage**	6452	MZD	Libya: unknown exact origin	E1 (HE585748)

References

1. Fritz, U. *et al.* Phenotypic plasticity leads to incongruence between morphology-based taxonomy and genetic differentiation in western Palaearctic tortoises (Testudo graeca complex; Testudines, Testudinidae). *Amphib.-Reptil.* **28,** 97–121 (2007).
2. Fritz, U. *et al.* Mitochondrial phylogeography of Testudo graeca in the Western Mediterranean: Old complex divergence in North Africa and recent arrival in Europe. *Amphib.-Reptil.* **30,** 63–80 (2009).
3. Vamberger, M., Corti, C., Stuckas, H. & Fritz, U. Is the imperilled spur-thighed tortoise (Testudo graeca) native in Sardinia? Implications from population genetics and for conservation. *Amphib.-Reptil.* **32,** 9–25 (2011).
4. Graciá, E. *et al.* The uncertainty of Late Pleistocene range expansions in the western Mediterranean: a case study of the colonization of south-eastern Spain by the spur-thighed tortoise, *Testudo graeca*. *J. Biogeogr.* **40,** 323–334 (2013).
5. Graciá, E. *et al.* Expansion after expansion: dissecting the phylogeography of the widely distributed spur-thighed tortoise, Testudo graeca (Testudines:Testudinidae). *Biological Journal of the Linnean Society* (In press). doi:10.1093/biolinnean/blx007

Supplementary Table S2 Pairwise F_{ST} estimates (below diagonal) and their associated *p* values (upper diagonal).

	**DO**	**LU**	**CR**	**BS**	**CH**	**MA**	**TE**	**MO**	**AC**	**SS**	**GU**	**TH**	**ZE**	**MO**	**MS**	**EK**
**DO**	0.000	0.001	0.001	0.001	0.001	0.001	0.001	0.001	0.001	0.001	0.001	0.001	0.001	0.001	0.001	0.001
**LU**	0.152	0.000	0.006	0.001	0.001	0.001	0.001	0.001	0.001	0.001	0.001	0.001	0.001	0.001	0.001	0.001
**CR**	0.154	0.064	0.000	0.003	0.001	0.001	0.001	0.001	0.001	0.001	0.001	0.001	0.001	0.001	0.001	0.001
**BS**	0.077	0.053	0.040	0.000	0.001	0.001	0.001	0.001	0.001	0.001	0.001	0.001	0.001	0.001	0.001	0.001
**CH**	0.077	0.083	0.099	0.048	0.000	0.001	0.001	0.001	0.001	0.001	0.001	0.001	0.001	0.001	0.001	0.001
**MA**	0.124	0.149	0.163	0.086	0.065	0.000	0.001	0.001	0.001	0.001	0.001	0.001	0.001	0.001	0.001	0.001
**TE**	0.093	0.140	0.164	0.071	0.073	0.137	0.000	0.001	0.001	0.001	0.001	0.001	0.001	0.001	0.001	0.001
**MO**	0.317	0.371	0.370	0.290	0.290	0.351	0.288	0.000	0.001	0.001	0.001	0.001	0.001	0.001	0.001	0.001
**AC**	0.190	0.306	0.284	0.208	0.234	0.283	0.251	0.201	0.000	0.013	0.027	0.001	0.001	0.001	0.012	0.056
**SS**	0.199	0.281	0.259	0.182	0.199	0.233	0.220	0.126	0.040	0.000	0.005	0.028	0.001	0.001	0.014	0.082
**GU**	0.200	0.310	0.278	0.193	0.223	0.288	0.242	0.198	0.026	0.036	0.000	0.002	0.001	0.015	0.406	0.149
**TH**	0.213	0.341	0.305	0.226	0.230	0.279	0.273	0.174	0.070	0.033	0.058	0.000	0.001	0.001	0.028	0.006
**ZE**	0.250	0.345	0.307	0.219	0.245	0.324	0.269	0.182	0.151	0.120	0.077	0.112	0.000	0.001	0.001	0.001
**MB**	0.219	0.344	0.323	0.241	0.249	0.306	0.282	0.261	0.069	0.071	0.026	0.066	0.137	0.000	0.072	0.001
**MS**	0.197	0.298	0.274	0.202	0.213	0.286	0.238	0.162	0.043	0.034	0.002	0.039	0.109	0.018	0.000	0.031
**EK**	0.230	0.304	0.270	0.201	0.225	0.294	0.249	0.199	0.028	0.022	0.018	0.058	0.117	0.073	0.037	0.000

Supplementary Table S3 Mean pairwise τ estimates calculated in BANANAS software.

	**DO**	**DO2**	**LU**	**CR**	**BS**	**CH**	**MA**	**TE**	**MO**	**AC**	**SS**	**KH**	**GU**	**TH**	**ZE**	**MO**	**MS**	**EK**
**DO**	0																	
**DO2**	0.67	0																
**LU**	1.03	1.44	0															
**CR**	0.89	1.47	0.48	0														
**BS**	0.41	1.17	0.22	0.32	0													
**CH**	0.44	1.17	0.47	0.34	0.21	0												
**MA**	0.77	1.55	0.49	0.57	0.20	0.34	0											
**TE**	0.47	1.21	0.64	0.38	0.14	0.16	0.21	0										
**MO**	0.83	1.11	1.35	1.12	0.96	1.11	1.13	1.29	0									
**AC**	0.82	1.38	1.62	1.20	1.15	1.15	1.44	1.23	0.60	0								
**SS**	0.64	1.17	1.46	1.06	0.63	0.55	0.88	0.85	0.31	0.27	0							
**KH**	0.74	1.17	1.55	0.89	1.18	0.92	1.28	1.14	0.69	0.19	0.39	0						
**GU**	0.58	1.37	1.60	0.99	0.74	0.72	1.06	0.97	0.41	0.31	0.18	0.19	0					
**TH**	0.88	1.54	1.50	1.26	1.12	0.77	1.12	1.36	0.94	0.90	0.36	0.33	0.44	0				
**ZE**	0.68	1.67	1.50	0.97	0.65	0.73	0.72	0.97	0.40	0.46	0.25	0.30	0.23	0.57	0			
**MB**	0.59	1.55	1.53	1.34	0.87	0.61	1.17	1.12	0.88	0.42	0.47	0.47	0.22	0.29	0.49	0		
**MS**	0.72	1.34	1.68	1.18	0.96	0.68	1.40	1.34	0.76	0.81	0.26	0.43	0.11	0.37	0.73	0.20	0	
**EK**	1.23	1.68	1.65	1.43	1.13	1.07	1.28	1.35	0.91	0.83	0.43	0.71	0.39	1.00	0.68	0.65	0.37	0

Supplementary Fig. S1 Results of the Bayesian clustering analyses in STRUCTURE[1]. The plot was obtained with STRUCTURE HARVESTER[2] and shows the magnitude of ΔK[3] according to *K* over 25 runs with 10 replicates.

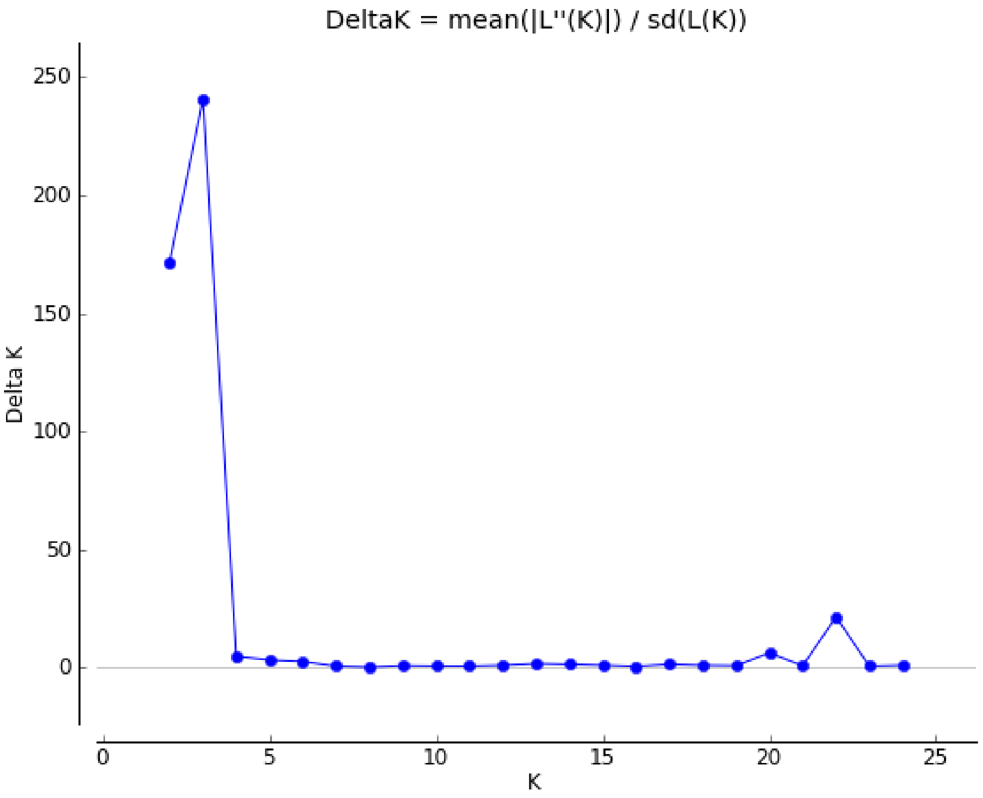

References:

1. Pritchard, J. K., Stephens, M. & Donnelly, P. Inference of population structure using multilocus genotype data. *Genetics* **155,** 945–959 (2000).
2. Earl, D. A. & vonHoldt, B. M. STRUCTURE HARVESTER: a website and program for visualizing STRUCTURE output and implementing the Evanno method. *Conserv. Genet. Resour.* **4,** 359–361 (2012).
3. Evanno, G., Regnaut, S. & Goudet, J. Detecting the number of clusters of individuals using the software structure: a simulation study. *Mol. Ecol.* **14,** 2611–2620 (2005).
